# Supplementary material for: Trends of frequency, mortality and risk factors among patients admitted with stroke from 2017 to 2019 to the medical ward at Kilimanjaro Christian Medical Centre hospital: a retrospective observational study
Source: BMJ Open. 2023 Jul 31;13(7):e071918. doi: 10.1136/bmjopen-2023-071918 (PMC10391824; doi:10.1136/bmjopen-2023-071918)
Supplement: Supplementary data [file bmjopen-2023-071918supp001.pdf]

Among all stroke patients identified, 229 (23.6%) died, while 458 (47.1%) of stroke patients stayed in hospital for more than thirty days **(Supplementary Table 1).**

Supplementary Table 1. Outcomes among stroke patients hospitalized at KCMC from 2017-2019.

Variables	Stroke		
	**Yes n (%) n=972**	**No n (%) n=7004**	***p*-value**
**Duration of Hospital stay (days)**			
0-7	202 (20.8)	1483 (21.2)	0.609
8-14	68 (7.0)	503 (7.2)	
15-21	110 (11.3)	872 (12.5)	
22-29	132 (13.6)	1009 (14.4)	
>30	458 (47.1)	3117 (44.5)	
Missing	2 (0.2)	20 (0.3)	
**Discharge status**			
Dead	229 (23.6)	1210 (17.3)	<0.001
Alive	742 (76.3)	5788 (82.6)	
Missing	1 (0.1)	6 (0.1)	
