# Supplementary material for: Trends of frequency, mortality and risk factors among patients admitted with stroke from 2017 to 2019 to the medical ward at Kilimanjaro Christian Medical Centre hospital: a retrospective observational study
Source: BMJ Open. 2023 Jul 31;13(7):e071918. doi: 10.1136/bmjopen-2023-071918 (PMC10391824; doi:10.1136/bmjopen-2023-071918)
Supplement: Supplementary data [file bmjopen-2023-071918supp002.pdf]

The number of stroke patients increased over the three years consecutively from 222, 292 to 458, revealing that between 2018 and 2019 number of stroke patients almost doubled **(Supplementary Figure 1).**

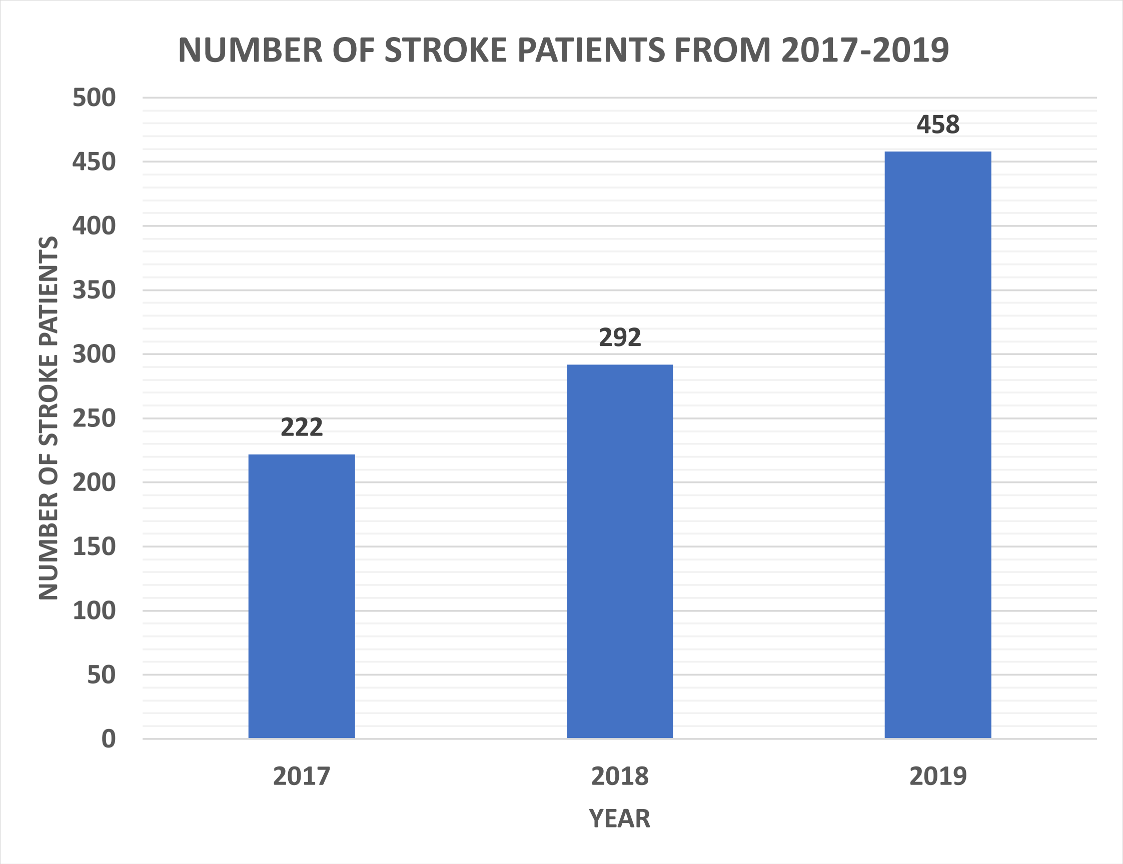

Supplementary Figure 1: Number of stroke admissions for 2017 to 2019
