# Supplementary material for: Trends of frequency, mortality and risk factors among patients admitted with stroke from 2017 to 2019 to the medical ward at Kilimanjaro Christian Medical Centre hospital: a retrospective observational study
Source: BMJ Open. 2023 Jul 31;13(7):e071918. doi: 10.1136/bmjopen-2023-071918 (PMC10391824; doi:10.1136/bmjopen-2023-071918)
Supplement: Supplementary data [file bmjopen-2023-071918supp003.pdf]

From 2017 to 2019 the number of deaths for non-stroke patients decreases from 506, 498 to 435 respectively. However, for the same three years the number of deaths for stroke patients increases from 60, 83 to 86 consecutively **(Supplementary Figure 2).**

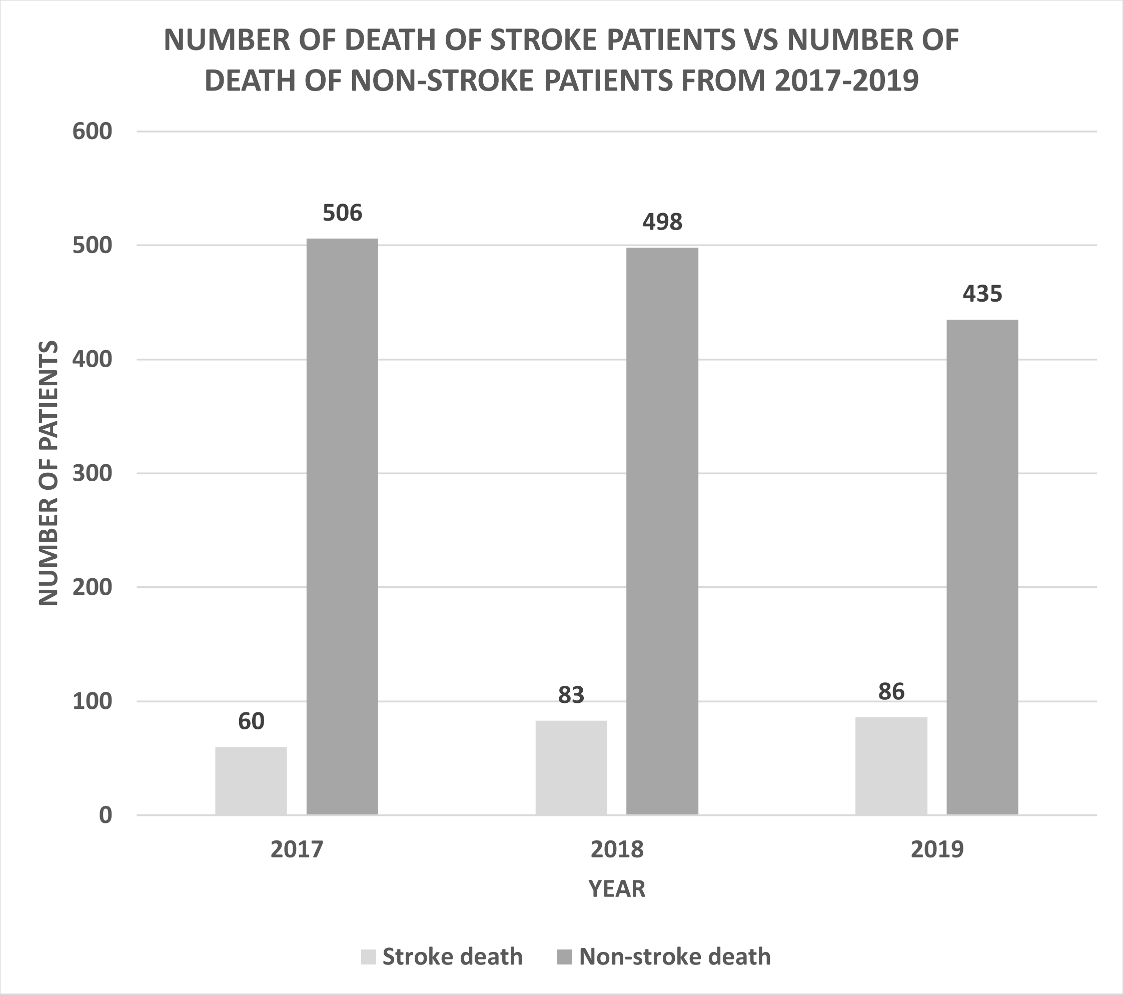

Supplementary Figure 2: Number of deaths for stroke patients Vs non-stroke patients for 2017-2019
