# Supplementary material for: Trends of frequency, mortality and risk factors among patients admitted with stroke from 2017 to 2019 to the medical ward at Kilimanjaro Christian Medical Centre hospital: a retrospective observational study
Source: BMJ Open. 2023 Jul 31;13(7):e071918. doi: 10.1136/bmjopen-2023-071918 (PMC10391824; doi:10.1136/bmjopen-2023-071918)
Supplement: Author's manuscript [file bmjopen-2023-071918.draft_revisions.pdf]

BMJ Open

BMJ Open is committed to open peer review. As part of this commitment we make the peer review history of every article we publish publicly available.

When an article is published we post the peer reviewers' comments and the authors' responses online. We also post the versions of the paper that were used during peer review. These are the versions that the peer review comments apply to.

The versions of the paper that follow are the versions that were submitted during the peer review process. They are not the versions of record or the final published versions. They should not be cited or distributed as the published version of this manuscript.

BMJ Open is an open access journal and the full, final, typeset and author-corrected version of record of the manuscript is available on our site with no access controls, subscription charges or pay-per-view fees (http://bmjopen.bmj.com).

If you have any questions on BMJ Open's open peer review process please email info.bmjopen@bmj.com

BMJ Open

Trends of frequency, mortality and risk factors among patients admitted with stroke from 2017 to 2019 to the medical ward at Kilimanjaro Christian Medical Centre hospital: a Retrospective Observational study

Journal:	*BMJ Open*
Manuscript ID	bmjopen-2023-071918
Article Type:	Original research
Date Submitted by the Author:	20-Jan-2023
Complete List of Authors:	Moshi, Baraka; Kilimanjaro Christian Medical University College, EPIDEMIOLOGY AND APPLIED BIOSTATISTICS; Kilimanjaro Clinical Research Institute<br>Yongolo, Nateiya ; Kilimanjaro Clinical Research Institute; Kilimanjaro Christian Medical University College<br>Biswaro, Sanjura Mandela; Kilimanjaro Clinical Research Institute; Kilimanjaro Christian Medical Centre<br>Maro, Hans; Kilimanjaro Christian Medical University College; Tanzania Health Promotion Support (THPS)<br>Linus, Sakanda; Kilimanjaro Christian Medical University College; Kilimanjaro Clinical Research Institute<br>Siebert, Stefan; University of Glasgow, Institute of Infection, Immunity and Inflammation; NHS Greater Glasgow and Clyde,<br>Nkenguye, William; Kilimanjaro Christian Medical University College<br>McIntosh, Emma; University of Glasgow Institute of Health and Wellbeing, Health Economics and Health Technology Assessment (HEHTA)<br>Shirima, Febronia; Kilimanjaro Clinical Research Institute; Kilimanjaro Christian Medical Centre<br>Njau, Rosalia E.; Kilimanjaro Clinical Research Institute; Kilimanjaro Christian Medical Centre<br>Andongolile, Alice A; Kilimanjaro Christian Medical University College; Kilimanjaro Clinical Research Institute<br>Mwanswila, Manasseh; Kilimanjaro Christian Medical Centre; Kilimanjaro Clinical Research Institute<br>Halliday, J; University of Glasgow<br>Krauth, Stefanie; School of Biodiversity, One Health and Veterinary Medicine, University Glasgow, Glasgow UK<br>Kilonzo, Kajiru; Kilimanjaro Christian Medical Centre, EPIDEMIOLOGY; Kilimanjaro Christian Medical University College, Medicine<br>Walker, Richard William; Newcastle University<br>Temu, Gloria August; Kilimanjaro Christian Medical Centre; Kilimanjaro Christian Medical University College<br>Mmbaga, Blandina; Kilimanjaro Clinical Research Institute; Kilimanjaro Christian Medical University College
Keywords:	Stroke medicine < INTERNAL MEDICINE, Stroke < NEUROLOGY, STROKE MEDICINE

I, the Submitting Author has the right to grant and does grant on behalf of all authors of the Work (as defined in the below author licence), an exclusive licence and/or a non-exclusive licence for contributions from authors who are: i) UK Crown employees; ii) where BMJ has agreed a CC-BY licence shall apply, and/or iii) in accordance with the terms applicable for US Federal Government officers or employees acting as part of their official duties; on a worldwide, perpetual, irrevocable, royalty-free basis to BMJ Publishing Group Ltd ("BMJ") its licensees and where the relevant Journal is co-owned by BMJ to the co-owners of the Journal, to publish the Work in this journal and any other BMJ products and to exploit all rights, as set out in our licence.

The Submitting Author accepts and understands that any supply made under these terms is made by BMJ to the Submitting Author unless you are acting as an employee on behalf of your employer or a postgraduate student of an affiliated institution which is paying any applicable article publishing charge ("APC") for Open Access articles. Where the Submitting Author wishes to make the Work available on an Open Access basis (and intends to pay the relevant APC), the terms of reuse of such Open Access shall be governed by a Creative Commons licence – details of these licences and which Creative Commons licence will apply to this Work are set out in our licence referred to above.

Other than as permitted in any relevant BMJ Author's Self Archiving Policies, I confirm this Work has not been accepted for publication elsewhere, is not being considered for publication elsewhere and does not duplicate material already published. I confirm all authors consent to publication of this Work and authorise the granting of this licence.

For peer review only

Trends of frequency, mortality and risk factors among patients admitted with stroke from 2017 to 2019 to the medical ward at Kilimanjaro Christian Medical Centre hospital: a Retrospective Observational study

*Baraka Moshi[2,3], Nateiya M Yongolo[2,3], Sanjura Mandela Biswaro[1,2], Hans Maro[3,7], Sakanda Linus[2,3], Stefan Siebert[8], William Nkenguye[3], Emma Sarah McIntosh[4], Febronia Shirima[2,3], Rosalia E. Njau[1,2], Alice A Andongolile[2,3], Manasseh Mwanswila[1,2], Jo E Halliday[3], Stefanie Krauth, Kajiru Kilonzo[1,3], Richard William Walker[6], Gloria August Temu[1,3], Blandina Theophil Mmbaga [2,3].

Affiliations:

[1]Kilimanjaro Christian Medical Centre, PO Box 3010, Moshi, Tanzania
[2]Kilimanjaro Clinical Research Institute, Box 2236, Moshi, Tanzania
[3]Kilimanjaro Christian Medical University College, Box 2240, Moshi, Tanzania
[4]Health Economics and Health Technology Assessment (HEHTA) School of Health and Wellbeing University of Glasgow, Glasgow United Kingdom.
[5]School of Biodiversity One Health and Veterinary Medicine, University of Glasgow, Glasgow United Kingdom.
[6]Population Health Sciences Institute, Newcastle University, Newcastle upon Tyne, United Kingdom
[7]Tanzania Health Promotion Support (THPS), Dar es salaam, Tanzania
[8]School of Infection & Immunity | College of Medical, Veterinary & Life Sciences University of Glasgow | 120 University Place | Glasgow G12 8TA

***Corresponding author Baraka Moshi email: bekamoshi0306@gmail.com**

Kilimanjaro Clinical Research Institute, Box 2236, Moshi, Tanzania +255719932222

For peer review only - http://bmjopen.bmj.com/site/about/guidelines.xhtml

Abstract

Objective: The burden of stroke has increased in recent years worldwide, particularly in low and middle-income countries. Study aims to determine the number of stroke admissions, and associated co-morbidities, to a referral hospital in Northern Tanzania.

Design: This was a retrospective Observational study.

Setting: The study was conducted at a tertiary referral hospital (KCMC) within the Northern zone of Tanzania.

Participants: The study included adults aged 18 and above, who were admitted to the medical wards from 2017 to 2019.

Outcome: Primary outcome was the proportion of Stroke patients admitted at medical ward and the secondary outcome was outcome of diagnosis.

Methods: We conducted a retrospective audit of medical record data from 2017 to 2019 for adult patients admitted in medical ward, at the KCMC. Data collected included demographic characteristics, previous history of stroke, and outcome of the admission. Factors associated with stroke were estimated using logistic regression.

Results: Among 8003 patients admitted during this time frame, 975 (12.2%) were stroke patients. Trends showed an increase in patients admitted with stroke over the 3 years from 222 (2.8%), 292 (3.6%) and 458 (5.7%) respectively. Of the stroke patients, 568 (58%) had hypertension while 167 (17%) had Diabetes mellitus. The proportion of stroke patients aged 18-45 years, increased from 2017 (n=28, 3.4%) to 2019 (n=40, 4.3%). Married individuals had higher odds of having strokes compared to single people (OR:3.6; CI:2.49,5.15). The in-hospital mortality related to stroke was 230 (23.6%) and females had higher odds of death as compared to male patients (OR:1.5; CI: 1.30,1.80).

Conclusion: The burden of stroke on individuals and health services is increasing over time, which reflects a lack of awareness and effective preventive measures. Prioritizing interventions directed towards the reduction of non-communicable diseases and associated complications such as stroke is urgently needed.

Keywords: Stroke, hospital, frequency, patients, trends, mortality

For peer review only

Strengths and limitations

- This was a hospital-based study rather than longitudinal community-based study, may be subjected to referral bias.
- The data were collected retrospectively from written documents which are prone to have missing information subjected to reporting bias.
- Our study contributes to the literature by providing crude estimates for stroke admissions, and co-occurrence with other NCDs.

KEY MESSAGE

What is already known on this topic

In Tanzania the Stroke incidence is well known, but still effort is needed by policy makers to monitor the incidence also primary intervention is needed to reduce the number of stroke admission.

What this study adds

This study aimed to identify the burden of stroke and co-morbid conditions in a consultant hospital

How this study might affect research, practice, or policy

By identifying the burden of Stroke will be crucial to increase general awareness of the growing burden of relevant stroke risk factors along with contemporary stroke trends and their consequences.

For peer review only

BACKGROUND

Stroke is defined by the World Health Organization as an acute, focal or diffuse, dysfunction of the brain, originating from vessels and lasting for a period longer than a day (1). Stroke is the second leading cause of death and main contributing cause of disability in adults a serious life-threatening non-communicable disease (NCDs) (2,3). Stroke leads to multiple physical and cognitive impacts such as difficulty in communication, memory loss, challenges with mobility, depression and sometimes essentially being bedridden (4).

Globally, an average of 1 in 4 adults will have a stroke during their lifetime. The burden of stroke in LMICs, including sub-Saharan Africa (SSA) has grown considerably in recent years (5). The incidence of a stroke in the past four decades (1970–2010) has increased by 100% in low and middle-income countries, but decreased by 42% in developed countries (6,7).

Interestingly, like most early hospital-based SSA studies, there was a predominance of male's admissions within the two earlier periods, but by the early 1990s, there were more females admitted to hospital with a stroke than males. If there really has been an increase in stroke over time, this could be explained by the increasing size of the population, increasing age of the population and potential changes in hospital usage. Most current reviews of stroke in patients especially in young adults and older people provide information about the etiology and diagnostic approach written mainly from a high-income perspective and do not take global differences into account (8,9).

Studies conducted in SSA have shown stroke has been increasing over the years as evidenced by observed proportions of admitted patients in tertiary hospitals, including at Kilimanjaro Christian

For peer review only - http://bmjopen.bmj.com/site/about/guidelines.xhtml

Medical Centre (KCMC). Interestingly, like most early hospital-based SSA studies, in a study from Ghana conducted over 3 decades there was a predominance of male admissions within the two earlier periods, but by the early 1990s, there were more females admitted to hospital with a stroke than males. In a similar study from KCMC over 4 decades there was a major increase in stroke admissions from the 1970s (less than 2 per year) to 2008 (more than 150 per year) (9). This could be explained by the increasing size of the population, increasing age of the population and potential changes in hospital usage. Most current reviews of stroke in patients, especially in young adults and older people, provide information about the etiology and diagnostic approach written mainly from a high-income perspective and do not take global differences into account (8,9)

The socioeconomic impact has undeniably increased over the past decade, and survival rates of stroke victims are going up. For most developed countries the incidence and mortality of stroke has been declining as a result of better hypertension control (2,10). However, for LMICs, due to lack of special attention and resources directed to prevention and control of risk factors for stroke, its associated morbidity and mortality is steadily increasing (11).

Despite both global and national plans to reduce the burden of NCDs still admission of Stroke patients is rising. There is limited information about Stroke trends in hospital admissions and outcomes among medical patients admitted to Kilimanjaro Christian Medical Centre.

This study aims to determine the number of stroke admissions, and associated co-morbidities, to a referral hospital in Northern Tanzania from 2017-to 2019.

For peer review only - http://bmjopen.bmj.com/site/about/guidelines.xhtml

Specific Objectives

1. To describe Socio-demographic characteristics and the association with stroke among patients admitted at KCMC/Northern zonal referral hospital 2017-2019
2. To determine numbers of inpatients recorded with stroke from 2017, 2018 and 2019.
3. To determine risk factors, comorbidities and outcomes associated with Stroke among patients hospitalized at KCMC from 2017-2019.

For peer review only

For peer review only - http://bmjopen.bmj.com/site/about/guidelines.xhtml

METHODS

Setting

KCMC is a tertiary hospital within the Northern zone of Tanzania. The hospital serves as a referral hospital for over 15 million people in 5 regions, namely Kilimanjaro, Arusha, Tanga, Singida and Manyara. Approximately 17,000 patients are seen at the hospital per month. The hospital has an in-patient capacity of 634 beds of which 100 are in the medical ward. The average daily admission rate for the medical department is 14 patients with an average length of stay of 8 days. The medical ward was selected because it's the main ward where stroke patients are admitted primarily regardless of their other comorbidities or complications.

Study design

This was a retrospective observational survey, involving review of medical record files for the years 2017, 2018 and 2019.

Study protocol

Patients' information was retrieved from hospital files stored in medical records and electronic patient data registry. Through using patient file numbers, files of patients admitted from 2017 to 2019 years were retrieved by medical record staff, and checked for eligibility. All eligible files were included for data to be extracted. Data was extracted using a prepared questionnaire, and entered into a redcap software after a quality check done by the study coordinator.

Trained data personnel captured information on a patient's unique number, age, gender, district of residence, level of education, occupation, religion, tribe, diagnosis, investigations done,

For peer review only - http://bmjopen.bmj.com/site/about/guidelines.xhtml

duration of hospital stay and number of admissions per year. Outcomes of admission such as death, referral to a higher facility and discharge home were also collected.

The diagnosis captured was the final diagnosis at the time of discharge, death or referral.

Study population

The study included adults aged 18 and above, who were admitted to the medical wards from 2017 to 2019 with stroke, or having stroke as an inpatient.

Data collection

Data were collected using a pre-formulated proforma which contained variables of interest to the study. A team of researchers obtained file numbers of all admissions to the medical ward for the years 2017 to 2019 from the medical records department. Using the file numbers obtained, team members retrieved the records from the hospital's medical records archive and electronic files systems. Data on demographic characteristics, diagnoses, treatments and outcomes of admissions were documented in the proforma. Diagnoses and comorbidities were extracted from the final discharge diagnoses recorded in the files of the patients.

For peer review only

Variable definitions

Outcomes

Primary outcome was the proportion of Stroke patients admitted at medical ward and the secondary outcome was outcome of diagnosis

Primary exposures

Social demographic characteristics (age gender, residence), Diseases and associated conditions

For peer review only - http://bmjopen.bmj.com/site/about/guidelines.xhtml

Eligibility criteria

Inclusion criteria

Adult's patients aged 18 and above, who were admitted to the medical wards from 2017 to 2019 with stroke.

Exclusion criteria

All patient under Intensive care unit.

Data analysis

The analysis was done using STATA version 15. Categorical variables were summarized using frequencies and percentages, while numerical variables were summarized by use of mean and standard deviation. Mortality was estimated by taking the total number of admissions divide by the total number of deaths at each diagnosed disease. Factors associated with stroke were estimated using multivariable logistic regression. Then the final model was developed by taking all exposures that were found to have a significant association with the outcome (stroke) and those that were considered as possible confounders in Univariate logistic regression were entered for the development of the final model. A p-value of less than 0.05 was considered as statistically significant.

Patient and public involvement

Patients and the public were not involved in the design and development of the entire study. The results of our study will be disseminated through open access publications.

For peer review only

RESULTS.

Socio demographics characteristics of stroke patients

A total of 8003 patients were admitted from January 2017 to December 2019 of whom 975 (12.2%) were stroke patients. Mean (SD) age of stroke patients was 57 (±32.6) years. Age of stroke patients ranges from 24 years to 90 years. The majority 827 (84.8%), of stroke patients resided in Kilimanjaro region with 317 (32.2%) being from Moshi rural district. Most, 675 (69.1%), were aged 60 years and above, 95 (9.7) were aged 45 years and below, more than half 503 (51.6%), were female and 384 (39.9%) were farmers **(Table 1)**.

Table 1. Socio-demographic characteristics among admitted stroke patients 2017-2019 (N=975)

**Variable**	**Stroke admission**			
	**(2017-2019)**	**2017**	**2018**	**2019**
**Age (years)**				
18-45	95(9.7)	28(12.6)	26(8.9)	40(4.7)
46-60	205(21.0)	44(19.8)	65(22.3)	96(21.0)
>60	675(69.1)	150(67.6)	201(68.8)	322(70.3)
Mean (SD)	57 (±32.6)			
**Sex (n=974)**				
Male	471(48.3)	101(45.5)	126(43.2)	243(53.1)
Female	503(51.6)	120(54.1)	166(56.8)	215(46.9)
**Marital status (n=538)**				
Single	33(3.4)	8(3.6)	17(5.8)	8(1.8)
Married	403(41.3)	115(51.8)	140(48.0)	148(32.3)
Divorced	16(1.6)	7(3.2)	6(2.1)	3(0.7)
Widowed	86(8.8)	24(10.8)	36(12.3)	26(5.7)
**Level of Education (n=78)**				

For peer review only - http://bmjopen.bmj.com/site/about/guidelines.xhtml

No Education	10(1.0)	7(3.2)	2(0.7)	1(0.2)
Primary	23(2.4)	8(3.6)	9(3.1)	6(1.3)
Secondary	2(0.2)	1(0.5)	0(0.0)	1(0.2)
University	43(4.4)	18(8.1)	11(3.8)	14(3.1)
**Residence**				
Kilimanjaro	827(84.8)	185(83.3)	249(85.3)	391(85.4)
Arusha	63(6.5)	17(7.7)	18(6.2)	28(6.1)
Tanga	10(1.0)	4(1.8)	2(0.7)	4(0.9)
Manyara	43 (4.4)	4(1.8)	18(6.2)	21(4.6)
Others	32(3.3)	12(5.4)	5(5.1)	14(3.1)
**Source of hospital payment**				
Cash	611(62.7)	148(66.7)	177(60.6)	285(62.2)
Insurance	343(35.2)	68(30.6)	110(37.7)	163(35.6)
Socio-welfare	7(0.7)	2(0.9)	1(0.3)	4(0.9)
Not reported	14(1.4)	4(1.8)	4(1.4)	6(1.3)

For peer review only

For peer review only - http://bmjopen.bmj.com/site/about/guidelines.xhtml

Comorbidities related to stroke, stroke rates and stroke mortality

Of the stroke patients, 568 (58%) had hypertension while 167 (17%) had Diabetes mellitus. Other comorbidities included pneumonia 241(21%), congestive cardiac failure 46 (4.7%), COPD/Asthma 6 (1%) and UTI 12 (1.2%) (**Table 2.)**

Table 2. Comorbidities associated with Stroke and its outcome in terms of inpatient mortality (N=975)

Variable	Stroke		Death
	Frequency	Percentage	n (%)
**Hypertension**	568	58.1	130(56.8)
**Diabetes Mellitus**	167	17.1	33(14.4)
**CCF**	46	4.7	14(6.1)
**Pneumonia**	241	24.8	110(48.0)
**CKD**	49	5.3	17(7.4)
**HIV/AIDS**	21	2.2.	7(3.1)
**AKI**	7	0.7	4(1.8)
**Asthma**	3	0.3	3(1.3)
**COPD**	3	0.3	1(0.4)
**DKA**	7	0.7	3(1.3)
**UTI**	12	1.2	4(1.8)

For peer review only - http://bmjopen.bmj.com/site/about/guidelines.xhtml

Among all stroke patients 229 (15.9%) died, majority 458 (12.8) of stroke patients stay in hospital for more than thirty days **(Table 3).**

Table 3. Outcomes among Stroke patients hospitalized at KCMC from 2017-2019.

**Variables**	**Stroke**		
	**Yes n (%)**	**No n (%)**	***p*-value**
**Duration of Hospital stay**			
0-7	202 (12.0)	1483 (88.0)	0.609
8-14	68 (11.9)	503 (88.1)	
15-21	110 (11.2)	872 (88.8)	
22-29	132 (11.6)	1009 (88.4)	
>30	458 (12.8)	3117 (87.2)	
**Discharge status**			
Dead	229 (15.9)	1210 (84.1)	<0.001
Alive	742 (11.4)	5788 (88.6)	

Factors associated with stroke, as compared to factors associated with other admissions

Among those admitted, older people aged above 60 years had significantly higher odds of having a stroke as compared to the age group 18-45 years old (OR:6.0;95CI:4.90, 7.63). Females had 20% higher odds of having strokes as compared to male (OR:1.2;95CI:1.05,1.37).

After adjusting to other factors old people aged above 60 years had significantly 4 times higher odds of having stroke as compared to the age group 18-45 years old (OR:4.2;95CI:3.27,5.28). However married individuals had significantly 60% higher odds of having stroke as compared to unmarried (OR:1.6;95CI:1.06,2.32). Overall prevalence of stroke was 12.2 with Confidence interval ranges from 0.115 to 0.129. **(Table 4).**

For peer review only - http://bmjopen.bmj.com/site/about/guidelines.xhtml

Table 4. Factors associated with Stroke patients admitted at KCMC compared to inpatients without stroke

Characteristics	Crude			Adjusted		
	Odds Ratio	95% CI	*p*-value	Odds Ratio	95% CI	*p*-value
**Age (years)**						
18-45	1			1		
46-60	3.0	2.34,3.86	<0.001	2.2	1.68,2.85	<0.001
>60	6.1	4.90,7.63	<0.001	4.2	3.27,5.28	<0.001
**Sex**						
Male	1			1		
Female	1.2	1.04,1.36	0.008	1.1	0.95,1.28	0.196
**Marital status**						
Single	1			1		
Married	3.5	2.49,5.15	<0.001	1.6	1.06,2.32	0.024
Divorced	1.7	0.92,3.14	0.096	0.8	0.43,1.57	0.546
Widowed	5.0	3.30,7.63	<0.001	1.6	1.04,2.61	0.034
**Level of Education**						
No Education	1			1		
Primary	0.5	0.21,1.04	0.066	0.9	0.36,2.09	0.749
Secondary	0.05	0.01,0.24	<0.001	0.2	0.04,1.08	0.063
Technical/University	0.4	0.21,0.95	0.036	0.9	0.38,1.96	0.722
**Hypertension**						
No	1			1		
Yes	4.1	3.61,4.62	<0.001	3.7	3.22,4.34	<0.001
**DM**						
No	1			1		

For peer review only - http://bmjopen.bmj.com/site/about/guidelines.xhtml

Yes	1.1	0.90,1.29	0.405	0.9	0.82,1.08	0.391
**CCF**						
No	1			1		
Yes	0.3	0.19,0.36	<0.001	0.2	0.11,0.21	<0.001
**CKD**						
No	1			1		
Yes	0.4	0.28,0.50	<0.001	0.3	0.21,0.39	<0.001
**HIV**						
No	1			1		
Yes	0.2	0.15,0.37	<0.001	0.4	0.27,0.67	<0.001

The number of Stroke patients keeps on increasing in three years consecutively from 222, 292 to 458, and between 2018 and 2019 number of Stroke patients almost doubled **(Figure 1).**

Trends showed an increase in proportions of patients admitted with stroke over the 3 years from 22.8% in 2017 to 30.0% in 2018 and 47.1% in 2019 **(Figure 2).**

While the proportion of death of non-stroke patients decreases from 35.2%, 34.6% to 30.2% as from 2017, 2018 and 2019 respectively**,** the proportion of stroke deaths increased, with 11.9%, 16.7% and 19.8% for 2017, 2018 and 2019, respectively (**Figure 3).**

From 2017 to 2019 the number of deaths for non-Stroke patients decreases from 506, 498 to 435 respectively. However, for the same three years the number of deaths for Stroke patients increases from 60, 83 to 86 consecutively **(Figure 4).**

For peer review only

For peer review only - http://bmjopen.bmj.com/site/about/guidelines.xhtml

Discussion

This was a retrospective study that looked at the number of adults with stroke diagnoses, out of all internal medicine (medical) admissions from 2017 to 2019. At 12.2% the proportion of admissions with stroke is high. The burden of stroke and its impact on society is increasing and it has been recognized as a condition that needs urgent attention in LMICs. In this study we aimed to identify the burden of stroke and co-morbid conditions in a consultant hospital. The proportion of overall stroke admissions for the 3 years was 22.8%, 30.0% and 47.0% for 2017, 2018 and 2019, respectively. This increase in the proportion of stroke admission had doubled by the end of the study period from 2017 to 2019. Significant changes in the proportion who died in hospital over the years of the study at 11.9%, 16.7% and 19.8%, respectively. We observed the common NCDs related to stroke were hypertension and diabetes while communicable diseases were pneumonia and HIV/AIDS.

Over the 3 years of the study the stroke admission rate increased 51.6%. This pattern of increasing stroke admissions over the years reflects a previous study conducted at KCMC comparing admission rates for stroke over the first 4 decades after KCMC opened in 1973 with less than 2 strokes per year in the 1970s increasing to over 150 stroke admissions per year in 2008(9). A previous study looking at patients aged 60 and over admitted to a hospital in Abeokuta, Nigeria, a hospital in Khartoum, Sudan and KCMC looking at patients aged 60 and over admitted over a 6-month period, showed that a quarter of the admissions at KCMC were due to stroke (12).

A recently published study from Zanzibar looking at hospital admissions with stroke over a 1-year period found very high rates of stroke admissions (13).

Our study showed a relationship between older age being more affected by more than one comorbidity, such as hypertension, diabetes, cardiac failure and pneumonia, and also being at higher risk of dying from stroke.

The rate of mortality in-hospital in this study was 23.5% which was lower compared to 27% reported in general medical inpatients admitted during the study period. The increased mortality of stroke patients was associated with comorbidities such as hypertension 96 (41.9%), pneumonia 86(41.8%), diabetes 26 (27.7%) and other NCDs. The case fatality rate was quite high compared to 16.2% found in Ethiopia (14). A UK which showed that the trend of stroke mortality decreased from 15.4% in 2008 to 9.8% in 2017(15). Mortality due to stroke in association with NCDs such as hypertension showed a significantly high death rate but the trend seems to be decreasing in our study through each year 2017,2018,2019 respectively which could be attributed to improved care and ease of access to medical services as well as an increase in access to health insurance services.

For peer review only - http://bmjopen.bmj.com/site/about/guidelines.xhtml

The most common risk factor identified was hypertension at 24%, consistent with other findings as uncontrolled hypertension is the most important risk factor for stroke both in developing and developed countries. This trend may reflect poor community awareness, health practices and access to healthcare including different patient related factors. A community-based study in the Hai District, which is near KCMC, demonstrated very high rates of undiagnosed hypertension in people aged 70 years and over, and that even in those who were diagnosed relatively few are on drug treatment, and in those who are on drug treatment even fewer were well controlled (16).

Diabetes mellitus is one of the major risk factors for the development of stroke. It was diagnosed as co-morbidity in 167 (17%) patients. In our study the prevalence of DM was higher compared to a study by Boot et.al which showed only 10% of stroke patients had DM (8). Ignorance of the risk factors and inability to manage such risk factors might be responsible for this effect.

Even when the patients understand the risk factors, they may not accept them to be the cause for stroke nor be able to afford the cost of medications. Additionally, since managing risk factors for stroke, such as hypertension, requires a longer period or may be lifelong; most patients fail to adhere and follow it properly (16). The above causes are likely to have contributed to the high rates of stroke among peoples with lower educational level, including rural urban people and farmers.

Extras- It was seen in the data recorded that a large number of stroke patients 32.5% came from Moshi rural areas and other districts of Kilimanjaro region which could be due to the fact that the majority of the elderly population live in rural areas.

Contributors: BM, BMB, NY, SB, RW and SL initial draft, revisions and data analysis. EM and BMB original idea of the paper, initiated the collaborative project and monitored data collection. KK, GT, MM, FS, AA and HM designed the study, revised the paper and works on methodology part. BM is the guarantor.

Funding: This work was supported by the National Institute for health and care Research award from United Kingdom (NIHR 17/63/35).

For peer review only - http://bmjopen.bmj.com/site/about/guidelines.xhtml

Competing Interests: None declared.

Data sharing statement: All data generated or analysed during this study are available from the Kilimanjaro Clinical Research Institute (KCRI) upon reasonable request from the corresponding author.

Ethics Statements

Patient Consent for Publication

Not applicable.

The ethical clearance was obtained from the Ethical Committee of the Kilimanjaro Christian Medical University College Research Ethics and Review Committee (CRERC) in Moshi **(KCMC/P. I/Vol.XI/2407),** The National Institute of Medical Research Review Committee (NatHREC) of the National Institute Medical Research (NIMR) in Tanzania. **(NIMR/HQ.R.8a/Vol.IX/3038)** and the Medical Veterinary and Life Sciences (MVLS) ethics committee at the University of Glasgow (UofG), UK **(200180100).** Permission was obtained from the President's Office, Regional Administration, and Local Government (POLARG), from the regional administrative secretary and regional medical officer office as well as the KCMC administration to conduct the study. No identifiable information was collected to maintain anonymity and the data were entered and stored into a secure database where only a few researchers with a password could access them.

Acknowledgments: We are grateful to all participants who participated in one way of this work and the data collection team

For peer review only

References

1. Sacco RL, Kasner SE, Broderick JP, Caplan LR, Connors JJ, Culebras A, et al. An updated definition of stroke for the 21st century: A statement for healthcare professionals from the American heart association/American stroke association. Stroke. 2013;44(7):2064–89.
2. Donkor ES. Stroke in the 21st Century: A Snapshot of the Burden, Epidemiology, and Quality of Life. Stroke Res Treat. 2018;2018.
3. Feigin VL, Stark BA, Johnson CO, Roth GA, Bisignano C, Abady GG, et al. Global, regional, and national burden of stroke and its risk factors, 1990-2019: A systematic analysis for the Global Burden of Disease Study 2019. Lancet Neurol. 2021;20(10):1–26.
4. Saengsuwan J, Suangpho P. Self-perceived and Actual Risk of Further Stroke in Patients with Recurrent Stroke or Recurrent Transient Ischemic Attack in Thailand. J Stroke Cerebrovasc Dis. 2019;28(3):632–9.
5. Alrabghi L, Alnemari R, Aloteebi R, Alshammari H, Ayyad M, Al Ibrahim M, et al. Stroke types and management. Int J Community Med Public Heal. 2018;5(9):3715.
6. Li Y, Li M, Wang M, Zhou Y, Chang J, Xian Y, et al. Acute cerebrovascular disease following COVID-19: a single center, retrospective, observational

For peer review only - http://bmjopen.bmj.com/site/about/guidelines.xhtml

study. Stroke Vasc Neurol. 2020;5(3):279–84.

7. Mekonen HH, Birhanu MM, Mossie TB, Gebreslassie HT. Factors associated with stroke among adult patients with hypertension in Ayder Comprehensive Specialized Hospital, Tigray, Ethiopia, 2018: A case-control study. PLoS One. 2020;15(2):1–10.

8. Boot E, Ekker MS, Putaala J, Kittner S, De Leeuw FE, Tuladhar AM. Ischaemic stroke in young adults: A global perspective. J Neurol Neurosurg Psychiatry. 2020;91(4):411–7.

9. Walker RW, Viney R, Green L, Mawanswila M, Maro VP, Gjertsen C, et al. Trends in stroke admissions to a Tanzanian hospital over four decades: A retrospective audit. Trop Med Int Heal. 2015;20(10):1290–6.

10. Lindmark A, Norrving B, Eriksson M. Socioeconomic status and survival after stroke - Using mediation and sensitivity analyses to assess the effect of stroke severity and unmeasured confounding. BMC Public Health. 2020;20(1):1–10.

11. Lee H, Nam YS, Lee KM. Development-assistance strategies for stroke in low- and middle-income countries. J Korean Med Sci. 2015;30(4):S139–42.

12. Kalaria RN, Akinyemi R, Ihara M. Stroke injury, cognitive impairment and vascular dementia. Biochim Biophys Acta - Mol Basis Dis [Internet]. 2016;1862(5):915–25. Available from:

For peer review only

For peer review only - http://bmjopen.bmj.com/site/about/guidelines.xhtml

http://dx.doi.org/10.1016/j.bbadis.2016.01.015

13. Mondal MBA, Hasan ATMH, Khan N, Mohammad QD. Prevalence and risk factors of stroke in Bangladesh: A nationwide population-based survey. eNeurologicalSci [Internet]. 2022;28(March):100414. Available from: https://doi.org/10.1016/j.ensci.2022.100414

14. Gadisa DA, Busawa GB, Gebremariam ET, Tefera GM, Belete KT, Taye GM, et al. Clinical Characteristics, Treatment Outcomes, and its Predictors Among Hospitalized Stroke Patients in Ambo University Referral Hospital, West Ethiopia: A Retrospective Hospital-Based Study. Vasc Health Risk Manag. 2020;16:591–604.

15. Rodríguez-Castro E, López-Dequit I, Santamaría-Cadavid M, Arias-Rivas S, Rodríguez-Yáñez M, Pumar JM, et al. Trends in stroke outcomes in the last ten years in a European tertiary hospital. BMC Neurol. 2018;18(1):1–10.

16. Dewhurst F, Dewhurst MJ, Gray WK, Aris E, Orega G, Howlett W, et al. The prevalence of neurological disorders in older people in Tanzania. Acta Neurol Scand. 2013;127(3):198–207.

For peer review only - http://bmjopen.bmj.com/site/about/guidelines.xhtml

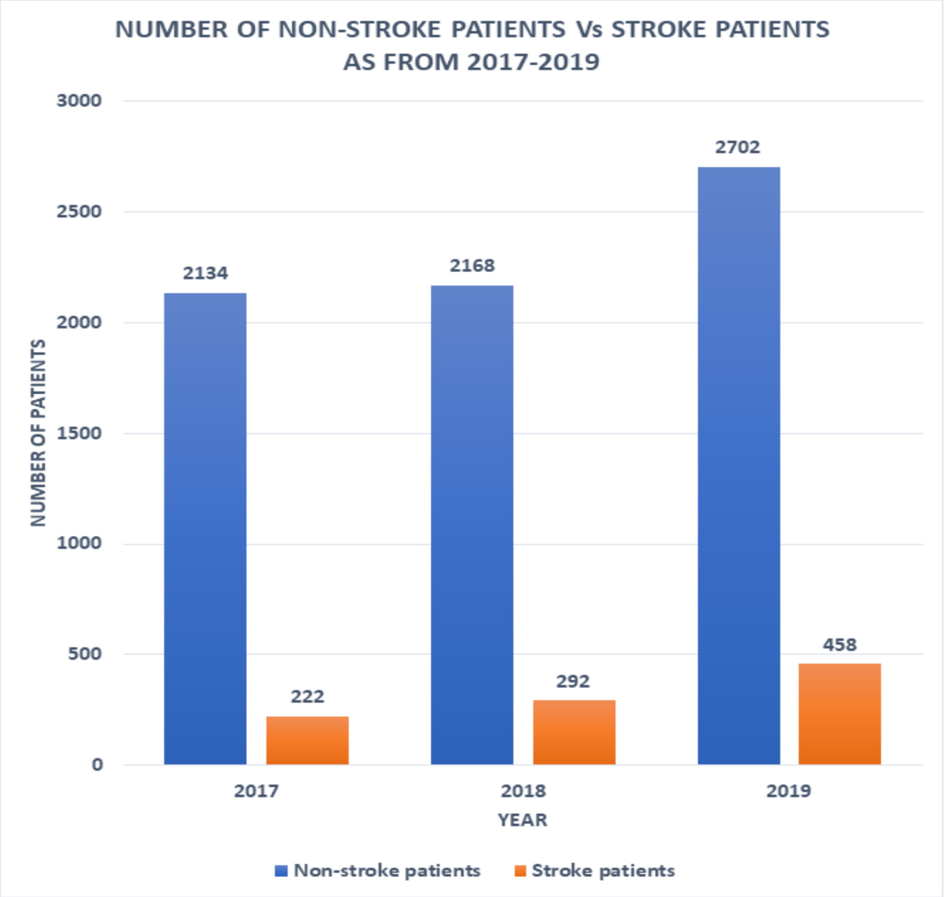

Figure 1: Number of Non-stroke patients Vs stroke patients as from 2017-2019

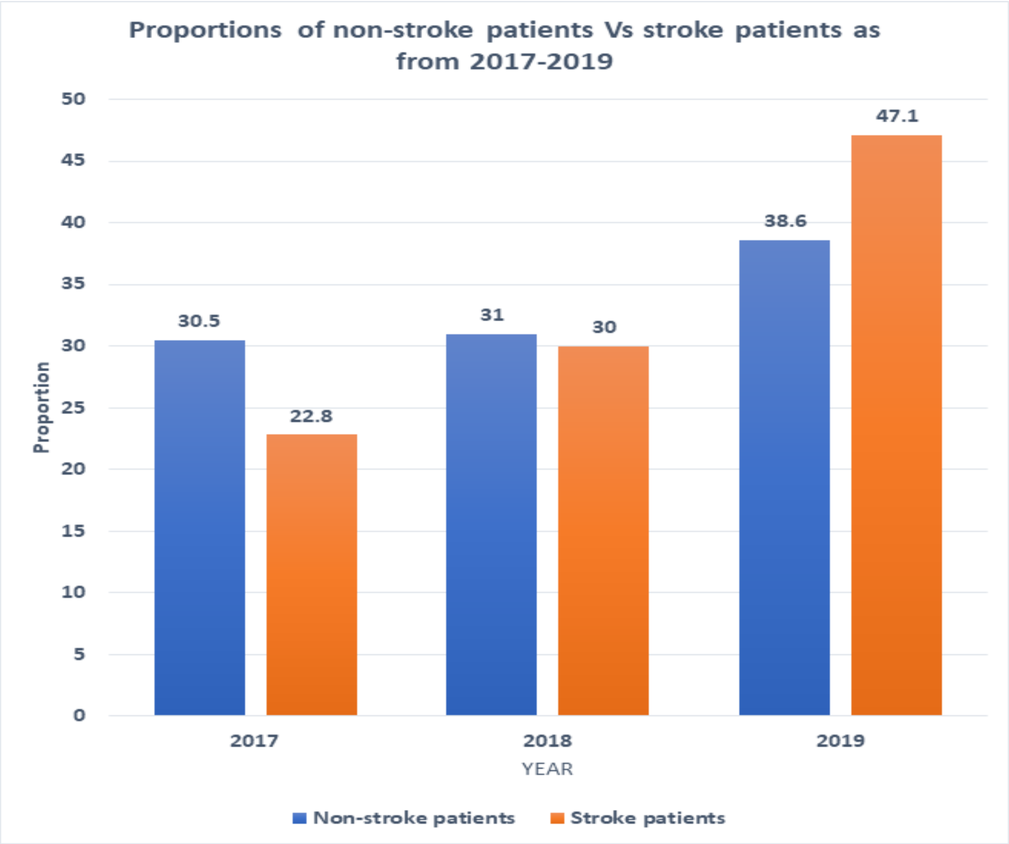

Figure 2: Proportions of non-stroke patients Vs stroke patients as from 2017-2019

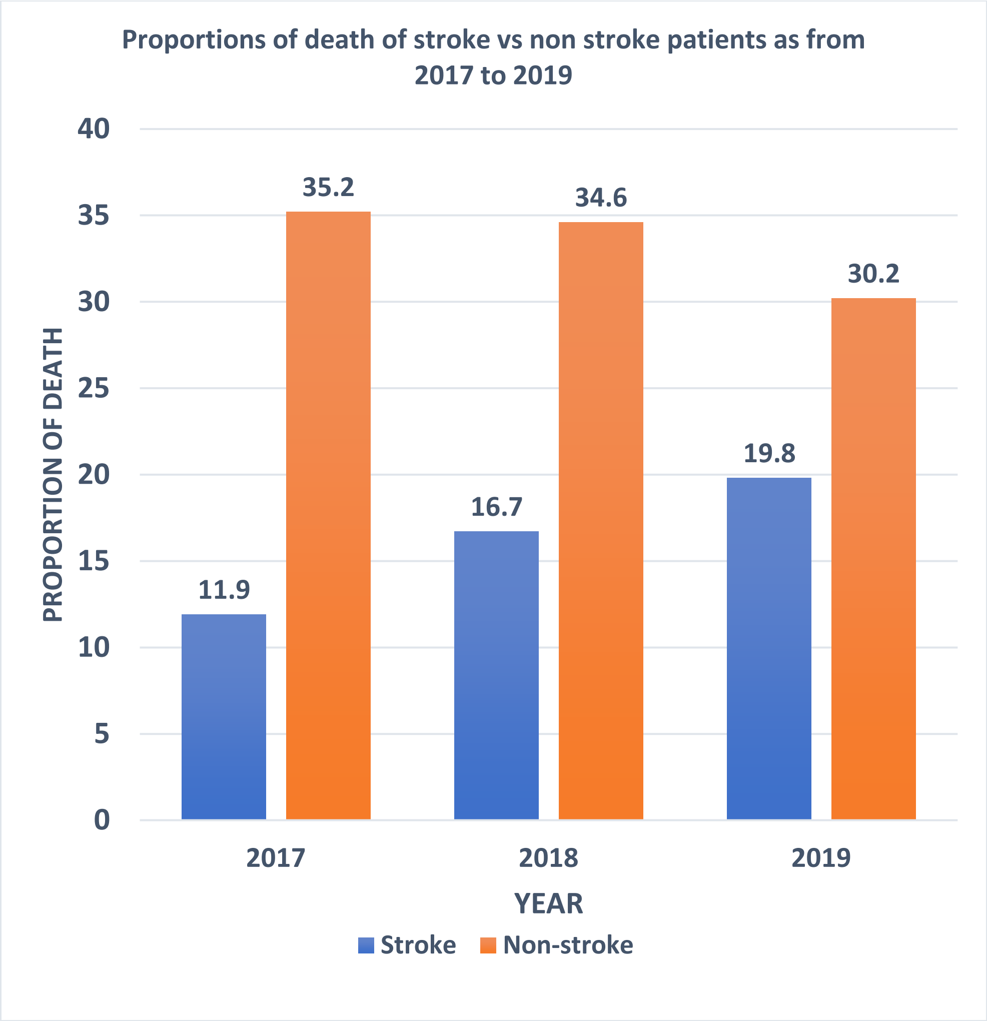

Figure 3. Proportion of deaths of stroke against non-stroke patients as from 2017-2019

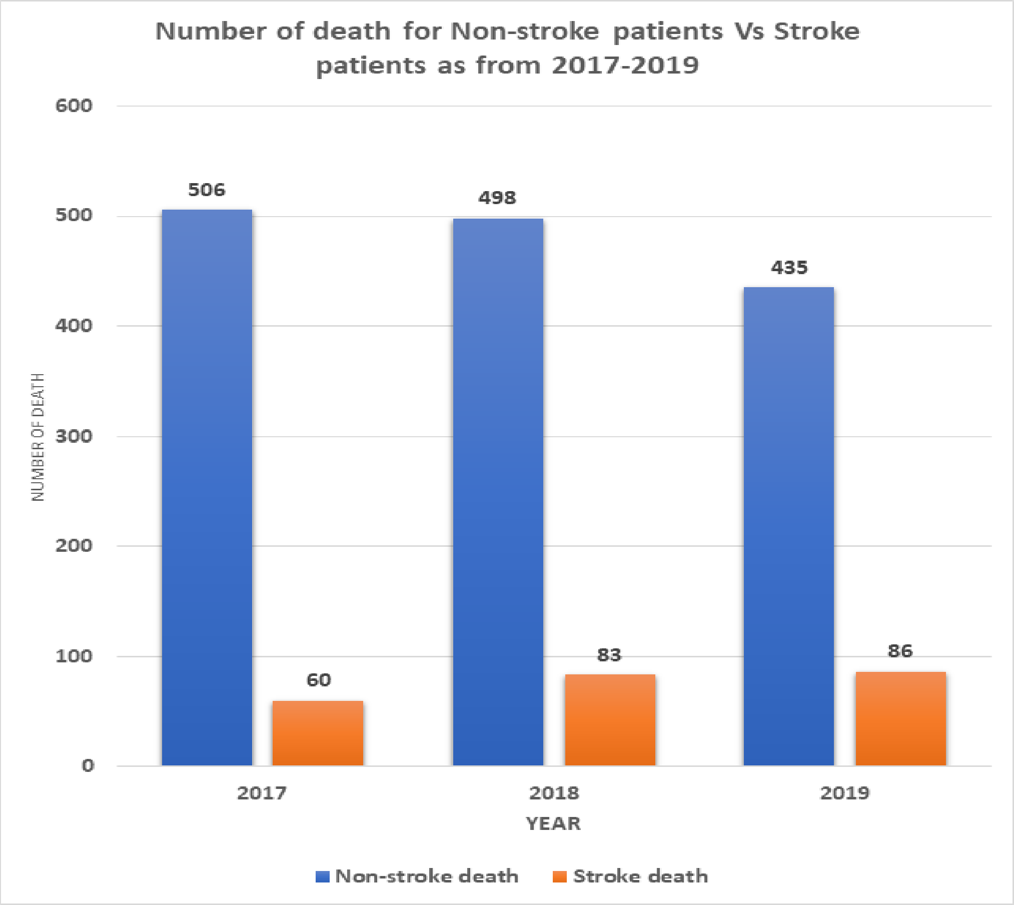

Figure 4: Number of deaths for non-stroke patients Vs stroke patients as from 2017-2019

STROBE Statement—Checklist of items that should be included in reports of ***cross-sectional studies***

	Item No	Recommendation	Page number
**Title and abstract**	1	(*a*) Indicate the study's design with a commonly used term in the title or the abstract	2
		(*b*) Provide in the abstract an informative and balanced summary of what was done and what was found	2-3
**Introduction**			
Background/rationale	2	Explain the scientific background and rationale for the investigation being reported	4-5
Objectives	3	State specific objectives, including any prespecified hypotheses	6
**Methods**			
Study design	4	Present key elements of study design early in the paper	7
Setting	5	Describe the setting, locations, and relevant dates, including periods of recruitment, exposure, follow-up, and data collection	7-8
Participants	6	(*a*) Give the eligibility criteria, and the sources and methods of selection of participants	8
Variables	7	Clearly define all outcomes, exposures, predictors, potential confounders, and effect modifiers. Give diagnostic criteria, if applicable	8
Data sources/ measurement	8*	For each variable of interest, give sources of data and details of methods of assessment (measurement). Describe comparability of assessment methods if there is more than one group	8
Bias	9	Describe any efforts to address potential sources of bias	8
Study size	10	Explain how the study size was arrived at	n/a
Quantitative variables	11	Explain how quantitative variables were handled in the analyses. If applicable, describe which groupings were chosen and why	8
Statistical methods	12	(*a*) Describe all statistical methods, including those used to control for confounding	8
		(*b*) Describe any methods used to examine subgroups and interactions	n/a
		(*c*) Explain how missing data were addressed	n/a
		(*d*) If applicable, describe analytical methods taking account of sampling strategy	n/a
		(*e*) Describe any sensitivity analyses	n/a
**Results**			
Participants	13*	(a) Report numbers of individuals at each stage of study—eg numbers potentially eligible, examined for eligibility, confirmed eligible, included in the study, completing follow-up, and analysed	n/a
		(b) Give reasons for non-participation at each stage	n/a
		(c) Consider use of a flow diagram	n/a
Descriptive data	14*	(a) Give characteristics of study participants (eg demographic, clinical, social) and information on exposures and potential confounders	10-15
		(b) Indicate number of participants with missing data for each variable of interest	n/a
Outcome data	15*	Report numbers of outcome events or summary measures	n/a
Main results	16	(*a*) Give unadjusted estimates and, if applicable, confounder-adjusted estimates and their precision (eg, 95% confidence interval). Make clear which confounders were adjusted for and why they were included	10-15

For peer review only - http://bmjopen.bmj.com/site/about/guidelines.xhtml

		(*b*) Report category boundaries when continuous variables were categorized	n/a
		(*c*) If relevant, consider translating estimates of relative risk into absolute risk for a meaningful time period	n/a
Other analyses	17	Report other analyses done—eg analyses of subgroups and interactions, and sensitivity analyses	n/a
**Discussion**			
Key results	18	Summarise key results with reference to study objectives	
Limitations	19	Discuss limitations of the study, taking into account sources of potential bias or imprecision. Discuss both direction and magnitude of any potential bias	16
Interpretation	20	Give a cautious overall interpretation of results considering objectives, limitations, multiplicity of analyses, results from similar studies, and other relevant evidence	10-16
Generalisability	21	Discuss the generalisability (external validity) of the study results	16
**Other information**			
Funding	22	Give the source of funding and the role of the funders for the present study and, if applicable, for the original study on which the present article is based	17

*Give information separately for exposed and unexposed groups.

Note: An Explanation and Elaboration article discusses each checklist item and gives methodological background and published examples of transparent reporting. The STROBE checklist is best used in conjunction with this article (freely available on the Web sites of PLoS Medicine at http://www.plosmedicine.org/, Annals of Internal Medicine at http://www.annals.org/, and Epidemiology at http://www.epidem.com/). Information on the STROBE Initiative is available at www.strobe-statement.org.

For peer review only

BMJ Open

Trends of frequency, mortality and risk factors among patients admitted with stroke from 2017 to 2019 to the medical ward at Kilimanjaro Christian Medical Centre hospital: a Retrospective Observational study

Journal:	*BMJ Open*
Manuscript ID	bmjopen-2023-071918.R1
Article Type:	Original research
Date Submitted by the Author:	01-Jun-2023
Complete List of Authors:	Moshi, Baraka; Kilimanjaro Christian Medical University College, EPIDEMIOLOGY AND APPLIED BIOSTATISTICS; Kilimanjaro Clinical Research Institute<br>Yongolo, Nateiya ; Kilimanjaro Clinical Research Institute; Kilimanjaro Christian Medical University College<br>Biswaro, Sanjura Mandela; Kilimanjaro Clinical Research Institute; Kilimanjaro Christian Medical Centre<br>Maro, Hans; Kilimanjaro Christian Medical University College; Tanzania Health Promotion Support (THPS)<br>Linus, Sakanda; Kilimanjaro Christian Medical University College; Kilimanjaro Clinical Research Institute<br>Siebert, Stefan; University of Glasgow, Institute of Infection, Immunity and Inflammation; NHS Greater Glasgow and Clyde,<br>Nkenguye, William; Kilimanjaro Christian Medical University College<br>McIntosh, Emma; University of Glasgow Institute of Health and Wellbeing, Health Economics and Health Technology Assessment (HEHTA)<br>Shirima, Febronia; Kilimanjaro Clinical Research Institute; Kilimanjaro Christian Medical Centre<br>Njau, Rosalia E.; Kilimanjaro Clinical Research Institute; Kilimanjaro Christian Medical Centre<br>Andongolile, Alice A; Kilimanjaro Christian Medical University College; Kilimanjaro Clinical Research Institute<br>Mwanswila, Manasseh; Kilimanjaro Christian Medical Centre; Kilimanjaro Clinical Research Institute<br>Halliday, J; University of Glasgow<br>Krauth, Stefanie; School of Biodiversity, One Health and Veterinary Medicine, University Glasgow, Glasgow UK<br>Kilonzo, Kajiru; Kilimanjaro Christian Medical Centre, EPIDEMIOLOGY; Kilimanjaro Christian Medical University College, Medicine<br>Walker, Richard William; Newcastle University<br>Temu, Gloria August; Kilimanjaro Christian Medical Centre; Kilimanjaro Christian Medical University College<br>Mmbaga, Blandina; Kilimanjaro Clinical Research Institute; Kilimanjaro Christian Medical University College
**Primary Subject Heading**:	Cardiovascular medicine

Secondary Subject Heading:	Cardiovascular medicine
Keywords:	Stroke medicine < INTERNAL MEDICINE, Stroke < NEUROLOGY, STROKE MEDICINE, GENERAL MEDICINE (see Internal Medicine)

SCHOLARONE™
Manuscripts

I, the Submitting Author has the right to grant and does grant on behalf of all authors of the Work (as defined in the below author licence), an exclusive licence and/or a non-exclusive licence for contributions from authors who are: i) UK Crown employees; ii) where BMJ has agreed a CC-BY licence shall apply, and/or iii) in accordance with the terms applicable for US Federal Government officers or employees acting as part of their official duties; on a worldwide, perpetual, irrevocable, royalty-free basis to BMJ Publishing Group Ltd ("BMJ") its licensees and where the relevant Journal is co-owned by BMJ to the co-owners of the Journal, to publish the Work in this journal and any other BMJ products and to exploit all rights, as set out in our licence.

The Submitting Author accepts and understands that any supply made under these terms is made by BMJ to the Submitting Author unless you are acting as an employee on behalf of your employer or a postgraduate student of an affiliated institution which is paying any applicable article publishing charge ("APC") for Open Access articles. Where the Submitting Author wishes to make the Work available on an Open Access basis (and intends to pay the relevant APC), the terms of reuse of such Open Access shall be governed by a Creative Commons licence – details of these licences and which Creative Commons licence will apply to this Work are set out in our licence referred to above.

Other than as permitted in any relevant BMJ Author's Self Archiving Policies, I confirm this Work has not been accepted for publication elsewhere, is not being considered for publication elsewhere and does not duplicate material already published. I confirm all authors consent to publication of this Work and authorise the granting of this licence.

For peer review only

Trends of frequency, mortality and risk factors among patients admitted with stroke from 2017 to 2019 to the medical ward at Kilimanjaro Christian Medical Centre hospital: a Retrospective Observational study

*Baraka Moshi[2,3], Nateiya M. Yongolo[2,3], Sanjura Mandela Biswaro[1,2], Hans Maro[3,7], Sakanda Linus[2,3], Stefan Siebert[8], William Nkenguye[3], Emma S. McIntosh[4], Febronia Shirima[2,3], Rosalia E. Njau[1,2], Alice A. Andongolile[2,3], Manasseh Mwanswila[1,2], Jo E.B. Halliday[3], Stefanie J. Krauth[4], Kajiru Kilonzo[1,3], Richard W. Walker[6], Gloria. A Temu[1,3], Blandina T. Mmbaga [2,3].

Affiliations:

[1]Kilimanjaro Christian Medical Centre, Moshi, Tanzania

[2]Kilimanjaro Clinical Research Institute, Moshi, Tanzania

[3]Kilimanjaro Christian Medical University College, Moshi, Tanzania

[4]School of Health and Wellbeing University of Glasgow, Glasgow, United Kingdom

[5]School of Biodiversity One Health and Veterinary Medicine, University of Glasgow, Glasgow, United Kingdom

[6]Population Health Sciences Institute, Newcastle University, Newcastle upon Tyne, United Kingdom

[7]Tanzania Health Promotion Support (THPS), Dar es salaam, Tanzania

[8]School of Infection & Immunity, University of Glasgow, Glasgow, United Kingdom

***Corresponding author Blandina T. Mmbaga: email:** b.mmbaga@kcri.ac.tz

Kilimanjaro Clinical Research Institute, Box 2236, Moshi, Tanzania +255768435116

For peer review only

List of abbreviations

**AIDS**	**Acquired Immune Deficiency Syndrome**
**AKI**	**Acute Kidney Injury**
**CCF**	**Congestive Cardiac Failure**
**CI**	**Confidence Interval**
**CKD**	**Chronic Kidney Disease**
**COPD**	**Chronic Obstructive Pulmonary Disease**
**DKA**	**Diabetic Ketoacidosis**
**DM**	**Diabetes Mellitus**
**HIV**	**Human Immune Virus**
**KCMC**	**Kilimanjaro Christian Medical Centre**
**KCRI**	**Kilimanjaro Clinical Research Institute**
**LMIC**	**Low and Middle Income Countries**
**NCD**	**Non-Communicable Diseases**
**OR**	**Odds Ratio**
**SSA**	**Sub-Saharan Africa**
**UTI**	**Urinary Tract Infections**
**WHO**	**World Health Organization**

For peer review only

For peer review only - http://bmjopen.bmj.com/site/about/guidelines.xhtml

Abstract

Objective: The burden of stroke has increased in recent years worldwide, particularly in low and middle-income countries. In this study we aim to determine the number of stroke admissions, and associated co-morbidities, to a referral hospital in Northern Tanzania.

Design: This was a retrospective observational study.

Setting: The study was conducted at a tertiary referral hospital, Kilimanjaro Christian Medical Centre (KCMC) within the Northern zone of Tanzania.

Participants: The study included adults aged 18 and above, who were admitted to the medical wards from 2017 to 2019.

Outcome: The primary outcome was the proportion of stroke patients admitted at medical ward at the Kilimanjaro Christian Medical Centre and the secondary outcome was clinical outcome such as mortality.

Methods: We conducted a retrospective audit of medical records from 2017 to 2019 for adult patients admitted to the medical ward at the KCMC. Data extracted included demographic characteristics, previous history of stroke, and outcome of the admission. Factors associated with stroke were investigated using logistic regression.

Results: Among 7976 patients admitted during between 2017 and 2019, 972 (12.2%) were stroke patients. Trends showed an increase in patients admitted with stroke over the 3 years with 222, 292 and 458 for 2017, 2018, 2019 respectively. Of the stroke patients, 568 (58.4%) had hypertension while 167 (17.2%) had Diabetes mellitus. The proportion of admitted patients with stroke patients aged 18-45 years, increased from 2017 (n=28, 3.4%) to 2019 (n=40, 4.3%). The in-hospital mortality related to stroke was 229 (23.6%) among 972 stroke patients and females had 50% higher odds of death as compared to male patients (OR:1.5; CI: 1.30,1.80).

Conclusion: The burden of stroke on individuals and health services is increasing over time, which reflects a lack of awareness on the cause of stroke and effective preventive measures. Prioritizing interventions directed towards the reduction of non-communicable diseases and associated complications, such as stroke, is urgently needed.

Keywords: stroke, hospital, frequency, patients, trends, mortality

For peer review only

For peer review only - http://bmjopen.bmj.com/site/about/guidelines.xhtml

Strengths and limitations

- Our study contributes to the literature by providing crude estimates for stroke admissions, and co-occurrence with other NCDs.
- This was a hospital-based study rather than longitudinal community-based study, may be subjected to referral bias.
- The data were collected retrospectively from written documents which are prone to have missing information subjected to reporting bias.

For peer review only

For peer review only - http://bmjopen.bmj.com/site/about/guidelines.xhtml

BACKGROUND

Stroke is defined by the World Health Organization as an acute, focal or diffuse, dysfunction of the brain, originating from vessels and lasting for a period longer than a day (1). Stroke is the second leading cause of death and main contributing cause of disability in adults worldwide (2,3). stroke leads to multiple physical and cognitive impacts such as difficulty in communication, memory loss, challenges with mobility, depression and sometimes being bedridden (4).

Globally, an estimated 1 in 4 adults will have a stroke during their lifetime. The burden of stroke in Low- and Middle-Income Countries (LMICs), including sub-Saharan Africa (SSA) has grown considerably in recent years (3). The incidence of stroke in the past four decades (1970–2010) has increased by 100% in LMICs, but decreased by 42% in developed countries (3,5). Most current reviews of stroke in patients, especially in young adults and older people provide information about the etiology and diagnostic approach written mainly from a high-income perspective and do not take global differences into account (6,7).

Studies conducted in SSA have shown stroke has been increasing over the years as evidenced by observed proportions of admitted patients in tertiary hospitals. Interestingly, like most early hospital-based SSA studies, in a study from Ghana conducted over 3 decades there was a predominance of male admissions within the two earlier periods, but by the early 1990s, there were more females admitted to hospital with a stroke than males (7). In a similar study from Kilimanjaro Christian Medical Centre (KCMC) over 4 decades, there was a major increase in stroke admissions from the 1970s (less than 2 per year) to 2008 (more than 150 per year) (7). The

For peer review only - http://bmjopen.bmj.com/site/about/guidelines.xhtml

changes made to primary health care in the last decade could be a major contributor to change in hospitals seeking behavior leading to rise in tertiary hospital referrals (8).

. For most developed countries the incidence and mortality of stroke has been declining as a result of better hypertension control (2,9). However, for LMICs, due to a lack of focused attention and limited resources directed to prevention and control of risk factors for stroke, its associated morbidity and mortality is steadily increasing (10).

In Tanzania, incidence of stroke is well known, however efforts are still needed by policy makers to monitor the incidence, increasing education awareness and develop better prevention interventions to reduce the number of stroke admissions. Identification of the burden of stroke will be crucial to increase general awareness of relevant risk factors along with stroke trends and their consequences.

Despite both global and national plans to reduce the burden of non-communicable diseases (NCDs), admission of stroke patients is still rising. There is limited recent information about trends in stroke hospital admissions and outcomes among medical inpatients admitted to KCMC.

The specific objectives of this study are as follows:

1. To describe the socio-demographic characteristics, comorbidities and their association with stroke among patients admitted at KCMC, Northern zonal referral hospital 2017-2019
2. To determine numbers of inpatients recorded with stroke for 2017, 2018 and 2019.
3. To determine outcomes of patients hospitalized with stroke at KCMC from 2017-2019.

For peer review only - http://bmjopen.bmj.com/site/about/guidelines.xhtml

METHODS

Setting

This study was conducted at KCMC, a zonal referral hospital within the Northern zone of Tanzania. The hospital serves as a referral hospital for over 15 million people in 5 regions, namely Kilimanjaro, Arusha, Tanga, Singida and Manyara. The healthcare system in Tanzania is a mix of both public and private services providers. The public healthcare system is organized into four levels: dispensaries, health centers, district hospitals, and referral hospitals. Dispensaries and health centers provide basic healthcare services, while district and referral hospitals offer more specialized care. In terms of hospital organization, Tanzanian hospitals, including KCMC, are typically organized into departments including internal medicine, general surgery, pediatrics, obstetrics and gynecology, oncology, orthopedic and others. The referral system goes from the dispensary level upward to the regional referral hospital, depending on the availability of required specialists and of medical equipment incl, CT and MRI scanners. The management of hospitals is overseen by the Ministry of Health. The department of Internal medicine specializes on prevention, diagnosis, and non-surgical treatment. The medical ward is the unit / department where patients are admitted and treated for medical conditions that require non-surgical care. Patients who are treated or admitted to the medical ward include those with chronic illnesses such as diabetes, hypertension, heart disease, lung disease, kidney disease and liver disease, as well as those with infectious diseases.

Approximately 17,000 patients are seen at KCMC per month. The hospital has an in-patient capacity of 634 beds of which 100 are in the medical ward. The average daily admission rate for the medical department is 14 patients with a bed occupancy rate of 99% and an average length

For peer review only - http://bmjopen.bmj.com/site/about/guidelines.xhtml

of stay of 8 days. The medical ward was the focus of this study because it is the main ward where stroke patients who do not require surgical intervention are admitted.

Study design

This was a retrospective observational survey, involving review of medical record files for individuals admitted to the medical ward at KCMC in the years 2017, 2018 and 2019.

Study protocol

Patients' information was retrieved from hospital files stored in medical records and the electronic patient data registry. Files of patients admitted from 2017 to 2019 were retrieved by medical record staff, and checked for eligibility. All eligible files were included for data extraction. Data was extracted using a prepared data extraction protocol, and entered into a REDCap (11) software. 10% of Files were double entered for quality control performed by the study coordinator.

Trained personnel captured information on a patient’s age, gender, district of residence, level of education, occupation, religion, tribe, diagnosis at the time of discharge, examinations and tests undertaken, duration of hospital stay, and number of admissions per year. Outcomes of admission such as death, referral to a higher facility, and discharge were also collected.

The diagnosis captured was the final diagnosis recorded in the medical record at the time of discharge, death or referral.

For peer review only - http://bmjopen.bmj.com/site/about/guidelines.xhtml

Study population

The study included adults aged 18 and above, who were admitted to the medical wards from 2017 to 2019 with stroke, or having stroke during their stay as an inpatient.

Data collection

Data were collected using a pre-formulated proforma which contained variables of interest to the study, as detailed above. A team of researchers obtained file numbers of all admissions to the medical ward for the years 2017 to 2019 from the medical records department. Using the file numbers obtained, team members retrieved the records from the hospital's medical records archive and electronic files systems. Data on demographic characteristics, diagnoses, treatments and outcomes of admissions were documented anonymously in the proforma. Diagnoses and comorbidities were extracted from the final discharge diagnoses recorded in the files of the patients.

Variable definitions

Outcomes

The primary outcome was the proportion of stroke patients admitted at medical ward and the secondary outcome was outcome of admission.

Secondary information

Social demographic characteristics (age, gender, marital status, level of education ethnicity, residence), source of hospital payment, discharge status, duration of hospital stay, diseases and associated conditions.

For peer review only - http://bmjopen.bmj.com/site/about/guidelines.xhtml

Eligibility criteria

Inclusion criteria

Adult patients aged 18 and above, who were admitted to the medical wards from 2017 to 2019 with stroke.

Exclusion criteria

All patients who were admitted directly to the Intensive Care Unit (ICU) and patients died at discharge point in Emergency medicine were excluded from this study because their information were not captured in the database.

Data analysis

The analysis was undertaken using STATA version 15 (Stata-Corp, College Station, TX, USA). Categorical variables were summarized using frequencies and percentages, while numerical variables were summarized by use of mean and standard deviation. Proportional mortality was estimated by taking the total number of admissions divided by the total number of deaths for each diagnosed disease. Factors associated with stroke were estimated using multivariable logistic regression. All exposures that were found to have a significant association with the outcome (stroke), and those that were considered as possible confounders in univariable logistic regression, were entered for the development of the final model. A p-value of less than 0.05 was considered as statistically significant.

Patient and public involvement

Patients and the public were not involved in the design and development of the study. The results of our study will be disseminated through open access publication, and publicized by other publicly accessible means.

For peer review only - http://bmjopen.bmj.com/site/about/guidelines.xhtml

RESULTS.

Socio demographics characteristics of stroke patients

A total of 7976 patients were admitted from January 2017 to December 2019, of whom 972 (12.2%) were stroke patients. Mean (standard deviation) age of stroke patients was 57 (±32.6) years. Age of stroke patients range from 24 years to 90 years. The majority 825 (84.5%), of stroke patients resided in Kilimanjaro region and 403 (41.3%) were married. Most, 673 (69.2%), were aged 60 years and above, while 94 (9.7) were aged 45 years and below. More than half of stroke patients, 501 (51.5%), were female. Moreover, the majority 610 (62.8%) of stroke patients use cash for hospital payment **(Table 1)**.

Table 1. Socio-demographic characteristics among admitted stroke patients 2017-2019 (N=972)

**Variable**	**stroke admission**			
	**(2017-2019)**	**2017**	**2018**	**2019**
	**n=972**	**n=222**	**n= 292**	**n= 458**
**Age (years)**	**n (%)**	**n (%)**	**n (%)**	**n (%)**
18-45	94 (9.7)	28 (12.6)	26 (8.9)	40 (4.7)
46-60	205 (21.1)	44 (19.8)	65 (22.3)	96 (21.0)
>60	673 (69.2)	150 (67.6)	201 (68.8)	322 (70.3)
**Mean (standard deviation)**	57 (±32.6)			
**Sex (n=971)**				
Male	470 (48.4)	101 (45.5)	126 (43.2)	243 (53.1)
Female	501 (51.6)	120 (54.1)	166 (56.8)	215 (46.9)
**Marital status**	**n=538**	**n=154**	**n=199**	**n=185**
Single	33 (6.1)	8 (5.2)	17 (8.5)	8 (4.3)
Married	403 (74.9)	115 (74.7)	140 (70.4)	148 (80.0)
Divorced	16 (3.0)	7 (4.5)	6 (3.0)	3 (1.6)

For peer review only - http://bmjopen.bmj.com/site/about/guidelines.xhtml

Widowed	86 (16.0)	24 (15.6)	36 (18.1)	26 (14.1)
**Residence**	n=966			
Kilimanjaro	825 (85.5)	185 (83.3)	249 (85.3)	391 (85.4)
Arusha	63 (6.5)	17 (7.7)	18 (6.2)	28 (6.1)
Tanga	10 (1.0)	4 (1.8)	2 (0.7)	4 (0.9)
Manyara	43 (4.5)	4 (1.8)	18 (6.2)	21 (4.6)
Others	25 (2.6)	12 (5.4)	5 (1.6)	14 (3.1)
**Source of hospital payment**				
Cash	610 (62.8)	148 (66.7)	177 (60.6)	285 (62.2)
Insurance	341 (35.1)	68 (30.6)	110 (37.7)	163 (35.6)
Socio-welfare	7 (0.7)	2 (0.9)	1 (0.3)	4 (0.9)
Not reported	14 (1.4)	4 (1.8)	4 (1.4)	6 (1.3)

Among all stroke patients identified, 229 (23.6%) died, while 458 (47.1%) of stroke patients stayed in hospital for more than thirty days **(Supplementary Table 1).**

Comorbidities related to stroke, stroke rates and stroke mortality

Of the stroke patients identified, 568 (58.4%) had hypertension while 167 (17.2%) had diabetes mellitus. Other comorbidities included congestive cardiac failure (CCF) 46 (4.7%), COPD (chronic obstructive pulmonary disease)/Asthma 6 (1%) and HIV/AIDS 21 (2.2%). However, some stroke patients had developed some complications whereby, 241 (24.8) of stroke patients had also pneumonia and among them 110 (48%) died and 12 (1.2%) had UTI (**Table 2).**

For peer review only

For peer review only - http://bmjopen.bmj.com/site/about/guidelines.xhtml

Table 2. Comorbidities and complications associated with stroke and its outcome in terms of inpatient mortality (N=972)

Variable	stroke		Death
	Frequency	Percentage	n (%)
**Hypertension**	568	58.4	130 (56.8)
**Diabetes Mellitus**	167	17.2	33 (14.4)
**CCF**	46	4.7	14 (6.1)
**Pneumonia**	241	24.8	110 (48.0)
**CKD**	49	5.0	17 (7.4)
**HIV/AIDS**	21	2.2.	7 (3.1)
**AKI**	7	0.7	4 (1.8)
**Asthma**	3	0.3	3 (1.3)
**COPD**	3	0.3	1 (0.4)
**DKA**	7	0.7	3 (1.3)
**UTI**	12	1.2	4 (1.8)

For peer review only

For peer review only - http://bmjopen.bmj.com/site/about/guidelines.xhtml

Factors associated with stroke, as compared to factors associated with other admissions

Among those admitted, older people aged above 60 years had significantly six times higher odds of having a stroke as compared to the age group 18-45 years old (OR: 6.0; 95CI: 4.90, 7.63). Females had 20% higher odds of having strokes as compared to male (OR: 1.2; 95CI: 1.05, 1.37). After adjusting for other factors people aged above 60 years had 4 times higher odds of having stroke as compared to the age group 18-45 years old (OR: 4.2; 95CI:3.27, 5.28). However married individuals had 60% higher odds of having stroke as compared to unmarried (OR:1.6;95CI:1.06,2.32) **(Table 3).**

Table 3. Factors associated with stroke patients admitted at KCMC compared to inpatients without stroke

Characteristics	Univariable			Multivariable		
	Odds Ratio	95% CI	*p*-value	Odds Ratio	95% CI	*p*- value
**Age (years)**						
18-45	1			1		
46-60	3.0	2.34, 3.86	<0.001	2.2	1.68, 2.85	<0.001
>60	6.1	4.90, 7.63	<0.001	4.2	3.27, 5.28	<0.001
**Sex**						
Male	1			1		
Female	1.2	1.04, 1.36	0.008	1.1	0.95, 1.28	0.196
**Marital status**						
Single	1			1		
Married	3.5	2.49, 5.15	<0.001	1.6	1.06, 2.32	0.024
Divorced	1.7	0.92, 3.14	0.096	0.8	0.43, 1.57	0.546
Widowed	5.0	3.30, 7.63	<0.001	1.6	1.04, 2.61	0.034
**Level of Education**						
No Education	1			1		
Primary	0.5	0.21, 1.04	0.066	0.9	0.36, 2.09	0.749

For peer review only - http://bmjopen.bmj.com/site/about/guidelines.xhtml

Secondary	0.05	0.01, 0.24	<0.001	0.2	0.04, 1.08	0.063
Technical/University	0.4	0.21, 0.95	0.036	0.9	0.38, 1.96	0.722
**Hypertension**						
No	1			1		
Yes	4.1	3.61, 4.62	<0.001	3.7	3.22, 4.34	<0.001
**DM**						
No	1			1		
Yes	1.1	0.90, 1.29	0.405	0.9	0.82, 1.08	0.391
**CCF**						
No	1			1		
Yes	0.3	0.19, 0.36	<0.001	0.2	0.11, 0.21	<0.001
**CKD**						
No	1			1		
Yes	0.4	0.28, 0.50	<0.001	0.3	0.21, 0.39	<0.001
**HIV**						
No	1			1		
Yes	0.2	0.15, 0.37	<0.001	0.4	0.27, 0.67	<0.001

For peer review only - http://bmjopen.bmj.com/site/about/guidelines.xhtml

Among all stroke patients identified, 229 (23.6%) died, while 458 (47.1%) of stroke patients stayed in hospital for more than thirty days **(Supplementary Table 1).**

Trends showed an increase in proportions of patients admitted with stroke compared to other conditions over the 3 years from 9.4% in 2017 to 11.9% in 2018 and 14.5% in 2019 **(Figure 1).**

The number of stroke patients increased over the three years consecutively from 222, 292 to 458, revealing that between 2018 and 2019 number of stroke patients almost doubled **(Supplementary Figure 1).**

While the proportion of deaths of non-stroke patients decreases from 35.2%, 34.6% to 30.2% as from 2017, 2018 and 2019 respectively**,** the proportion of stroke deaths increased, with 11.9%, 16.7% and 19.8% for 2017, 2018 and 2019, respectively (**Figure 2).**

From 2017 to 2019 the number of deaths for non-stroke patients decreases from 506, 498 to 435 respectively. However, for the same three years the number of deaths for stroke patients increases from 60, 83 to 86 consecutively **(Supplementary Figure 2).**

For peer review only

For peer review only - http://bmjopen.bmj.com/site/about/guidelines.xhtml

Discussion

This retrospective study looked at the number of adults with stroke diagnoses, out of all internal medicine (medical) admissions from 2017 to 2019 in KCMC, Tanzania. At 12.2%, the proportion of admissions with stroke is high as compared to the finding from the study in China looking on the prevalence and it related risk factors whereby the overall prevalence of stroke was 3.1% (12). The burden of stroke and its impact on society is increasing and it has been recognized as a condition that needs urgent attention in LMICs. In this study we aimed to identify the burden of stroke and co-morbid conditions in a consultant hospital. The proportion of overall stroke admissions for the 3 years, each year was 22.8%, 30.0% and 47.0% for 2017, 2018 and 2019, respectively. This increase in the proportion of stroke admissions had doubled by the end of the study period. There were significant changes in the proportion of stroke patients who died in hospital over the years of the study at 11.9%, 16.7% and 19.8%, respectively. This rate is however lower than a study conducted in a Burkina Faso which had mortality rate of stroke patients 39%, respectively, 37.6% in male and 41.6% in female where the mortality risk factors were the same. This disparity could be due to low access to health facilities for routine medical checkup which could prevent the adverse outcomes of stroke and inadequate infrastructure and trained personnel with low level of care given in those established facilities in Burkina Fuso(13). We observed the common NCDs related to stroke were hypertension and diabetes while communicable disease was HIV/AIDS.

Over the 3 years of the study the stroke admission rate increased by 51.6%. This pattern of increasing stroke admissions over the years reflects a previous study conducted at KCMC comparing admission rates for stroke over the first 4 decades after KCMC opened in 1973 with less than 2 strokes per year in the 1970s increasing to over 150 stroke admissions per year in 2008 (7). The increase in stroke admissions over time could partly be explained by the increasing size of the population, increasing age of the population and potential changes in hospital usage. A previous study looking at patients aged 60 and over admitted to a hospital in Abeokuta, Nigeria, a hospital in Khartoum, Sudan and KCMC looking at patients aged 60 and over admitted over a 6-month period, showed that a quarter of the admissions at KCMC were due to stroke (14).

For peer review only - http://bmjopen.bmj.com/site/about/guidelines.xhtml

A recently published study from Zanzibar looking at hospital admissions with stroke over a 1-year period found very high rates of stroke admissions whereby, among 720 participants overall, 463 (64.3%) participants were experiencing a first-ever stroke, and 223 (31.0%) a recurrent stroke (15).

The rate of in-hospital mortality in this study was 23.6% which was lower compared to the 27% reported in general medical inpatients admitted during the study period. The increased mortality of stroke patients was associated with comorbidities such as hypertension 96 (41.9%), diabetes 26 (27.7%) and other NCDs. The case fatality rate was higher than the rate of 16.2% found in Ethiopia (16).

In a European tertiary referral hospital study stroke mortality decreased from 15.4% in 2008 to 9.8% in 2017 (8).

Mortality due to stroke in association with NCDs such as hypertension was high but the trend seems to be decreasing in our study through each year, 2017,2018,2019 respectively, which could be attributed to improved care and ease of access to medical services as well as an increase in access to health insurance services.

The most common risk factor for stroke identified in our study was hypertension, with a total prevalence of 24% among all admitted patients and 58.4% among stroke patients had also hypertension. This finding is consistent with other findings as uncontrolled hypertension is the most important risk factor for stroke both in developing and developed countries. This trend may reflect poor community awareness, health practices and access to healthcare including different patient related factors. A community-based study in the Hai District, which is near KCMC, demonstrated very high rates of undiagnosed hypertension in people aged 70 years and over, and that even in those who were diagnosed relatively few were on drug treatment, and in those who were on drug treatment even fewer were well controlled (7).

Diabetes mellitus is one of the major risk factors for the development of stroke. It was identified as a co-morbidity in 167 (17.2%) patients. In our study the prevalence of DM was higher compared to a study by Boot et.al which showed only 10% of stroke patients had DM (6).

For peer review only - http://bmjopen.bmj.com/site/about/guidelines.xhtml

Ignorance of the risk factors and inability to manage such risk factors might be responsible for this effect.

A serious and common complication of stroke is pneumonia, stroke-associated pneumonia is frequently caused by aspiration this is similar findings from our study revealed that the 24.8% among stroke patients end up having pneumonia whereby, 110 (48%) died, the same to the findings of the study by Grossmann. Pneumonia occurs in 4%-10% of patients experiencing a stroke, making it one of the most problematic complications, associating it with a high mortality rate (17).

Even when the patients understand the risk factors, they may not accept them to be the cause for stroke nor be able to afford the cost of medications. Additionally, since managing risk factors for stroke, such as hypertension, requires a longer period or may be lifelong; most patients fail to adhere and follow advice properly (18). The above causes are likely to have contributed to the high rates of stroke among people with lower educational level, including rural farmers.

A previous community-based stroke incidence study from 2003 – 2006, which was conducted in the rural Hai District, near to KCMC, and an urban area in Dar es Salaam, demonstrated very high stroke incidence rates, comparable to African Americans in Manhattan, New York, USA (19). This study involved active case findings and those who died before they were seen were picked up on verbal autopsy. Many patients do not go to hospital following their stroke and there is usually a significant delay so hospital statistics will underestimate the community burden.

Sudy limitations

This was a hospital-based study rather than longitudinal community-based study, may be subjected to referral bias whereby patients referred to KCMC hospital may have some other complications which differ from patients treated to other health facilities. The type of data analyzed in this study does not allow for

For peer review only - http://bmjopen.bmj.com/site/about/guidelines.xhtml

a difference between risk factors, comorbidities and complications, as we have no data on the order of these factors, we therefore present all factors as associated.

The data were collected retrospectively from written documents which are prone to have missing information subjected to reporting bias.

Strengths of the study

Our study contributes to the literature by providing crude estimates for stroke admissions, and co-occurrence with other NCDs.

Conclusion

The burden of stroke on individuals and health services is increasing over time, which reflects a lack of awareness on the both modifiable and non-modifiable cause of stroke and effective preventive measures. Prioritizing interventions directed towards the reduction of non-communicable diseases and associated complications such as stroke is urgently needed.

Contributors: BM, BTM, NY, SB, RW, KK, SK and SL initial draft, revisions and data analysis. and GT, BTB had the original idea for the research project, initiated the collaborative project and monitored data collection. BM, KK, NY, SK, SB, JH, EM, AA, WN, RW, MM, SS, FS, GT, RN, BTM, HM and SL designed the study, revised the paper and worked on methodology component. BM is the guarantor.

Funding: This work was supported by the National Institute for Health and Care Research award from United Kingdom (NIHR 17/63/35).

Competing Interests: None declared.

Data sharing statement: All data generated or analysed during this study are available from the Kilimanjaro Clinical Research Institute (KCRI) upon reasonable request from the corresponding author.

For peer review only - http://bmjopen.bmj.com/site/about/guidelines.xhtml

Ethics Statements

Patient Consent for Publication

Not applicable.

The ethical clearance was obtained from the Ethical Committee of the Kilimanjaro Christian Medical University College Research Ethics and Review Committee (CRERC) in Moshi **(KCMC/P. I/Vol.XI/2407),** The National Institute of Medical Research Review Committee (NatHREC) of the National Institute Medical Research (NIMR) in Tanzania. **(NIMR/HQ.R.8a/Vol.IX/3038)** and the Medical Veterinary and Life Sciences (MVLS) ethics committee at the University of Glasgow (UofG), UK **(200180100).** Permission was obtained from the President's Office, Regional Administration, and Local Government (POLARG), from the regional administrative secretary and regional medical officer office as well as the KCMC administration to conduct the study. No identifiable information was collected to maintain anonymity and the data were entered and stored into a secure database where only a few researchers with a password could access them.

Acknowledgments: We are grateful to all participants who participated in one way of this work and the data collection team

For peer review only

References

1. Sacco RL, Kasner SE, Broderick JP, Caplan LR, Connors JJ, Culebras A, et al. An updated definition of stroke for the 21st century: A statement for healthcare professionals from the American heart association/American stroke association. stroke. 2013;44(7):2064–89.

2. Donkor ES. stroke in the 21st Century: A Snapshot of the Burden, Epidemiology, and Quality of Life. stroke Res Treat. 2018;2018.

3. Feigin VL, Stark BA, Johnson CO, Roth GA, Bisignano C, Abady GG, et al. Global, regional, and national burden of stroke and its risk factors, 1990-2019: A systematic analysis for the Global Burden of Disease Study 2019. Lancet Neurol. 2021;20(10):1–26.

4. Saengsuwan J, Suangpho P. Self-perceived and Actual Risk of Further stroke in Patients with Recurrent stroke or Recurrent Transient Ischemic Attack in Thailand. J stroke Cerebrovasc Dis. 2019;28(3):632–9.

5. Mekonen HH, Birhanu MM, Mossie TB, Gebreslassie HT. Factors associated with stroke among adult patients with hypertension in Ayder Comprehensive Specialized Hospital, Tigray, Ethiopia, 2018: A case-control study. PLoS One. 2020;15(2):1–10.

6. Boot E, Ekker MS, Putaala J, Kittner S, De Leeuw FE, Tuladhar AM. Ischaemic stroke in young adults: A global perspective. J Neurol Neurosurg Psychiatry. 2020;91(4):411–7.

7. Walker RW, Viney R, Green L, Mawanswila M, Maro VP, Gjertsen C, et al. Trends in stroke admissions to a Tanzanian hospital over four decades: A retrospective audit. Trop Med Int Heal. 2015;20(10):1290–6.

8. Rodríguez-Castro E, López-Dequit I, Santamaría-Cadavid M, Arias-Rivas S, Rodríguez-Yáñez M, Pumar JM, et al. Trends in stroke outcomes in the last ten years in a European tertiary

For peer review only - http://bmjopen.bmj.com/site/about/guidelines.xhtml

hospital. BMC Neurol. 2018;18(1):1–10.

9. Lindmark A, Norrving B, Eriksson M. Socioeconomic status and survival after stroke - Using mediation and sensitivity analyses to assess the effect of stroke severity and unmeasured confounding. BMC Public Health. 2020;20(1):1–10.

10. Lee H, Nam YS, Lee KM. Development-assistance strategies for stroke in low- and middle-income countries. J Korean Med Sci. 2015;30(4): S139–42.

11. Harris PA, Taylor R, Thielke R, Payne J, Gonzalez N, Conde JG. Research electronic data capture (REDCap)—A metadata-driven methodology and workflow process for providing translational research informatics support. J Biomed Inform. 2009 Apr;42(2):377–81.

12. Yi X, Luo H, Zhou J, Yu M, Chen X, Tan L, et al. Prevalence of stroke and stroke related risk factors: a population based cross sectional survey in southwestern China. BMC Neurol. 2020 Dec;20(1):5.

13. Dabilgou AA, Dravé A, Kyelem JMA, Ouedraogo S, Napon C, Kaboré J. Frequency and Mortality Risk Factors of Acute Ischemic Stroke in Emergency Department in Burkina Faso. Stroke Res Treat. 2020 Jun 11; 2020:1–7.

14. R O Akinyemi, I M H Izzeldin, C L Dotchin, W K. Gray, O Adeniji, O A. Seidi, J J Mwakisambwe, C J Mhina, F Mutesi, H Z. Msechu, K A. Mteta, M A.M. Ahmed, S H.M. Hamid, N A.A. Abuelgasim, S A.A. Mohamed, A Y O Mohamed, F Adesina, M Hamzat, T Olunuga, V P Maro, R W Walker. Contribution of Non-communicable Diseases to Medical Admissions of Elderly Adults in Africa: A Prospective, Cross-Sectional Study in Nigeria, Sudan and Tanzania. Journal of the American Geriatrics Society 2014 Aug;62(8):1460-6.

For peer review only - http://bmjopen.bmj.com/site/about/guidelines.xhtml

15. Jutta Jorgensen, Dirk Christensen, Karoline Nielsen, Muhammad Khan, Halima Sadiq, Ahmed Jusabani, and Richard Walker. stroke in Zanzibar - incidence and characteristics of strok in Zanzibar – a hospital-based prespective study in a low-income island population. Frontiers in Neurology 2022 (in press).

16. Gadisa DA, Busawa GB, Gebremariam ET, Tefera GM, Belete KT, Taye GM, et al. Clinical Characteristics, Treatment Outcomes, and its Predictors Among Hospitalized Stroke Patients in Ambo University Referral Hospital, West Ethiopia: A Retrospective Hospital-Based Study. Vasc Health Risk Manag. 2020; 16:591–604.

17. Grossmann I, Rodriguez K, Soni M, Joshi PK, Patel SC, Shreya D, et al. Stroke and Pneumonia: Mechanisms, Risk Factors, Management, and Prevention. Cureus [Internet]. 2021 Nov 26 [cited 2023 May 14]; Available from: https://www.cureus.com/articles/78536-stroke-and-pneumonia-mechanisms-risk-factors-management-and-prevention.

18. M J Dewhurst, F Dewhurst, W K Gray, P Chaote, G P Orega. R W Walker. The high prevalence of hypertension in rural-dwelling Tanzanian older adults and the disparity between detection, treatment and control: A rule of sixths? Journal of Human Hypertension 2013 Jun; 27 (6): 374-80. doi:10.1038/jhh.2012.59. PMID: 23235367.

19. R W Walker, D Whiting, N Unwin, F Mugusi, M Swai, E Aris, A Jusabani, G Kabadi, W K. Gray, M Lewanga, G Alberti. stroke incidence in rural and urban Tanzania: a prospective community-based study. Lancet Neurology 2010; (9): 786-792.

For peer review only - http://bmjopen.bmj.com/site/about/guidelines.xhtml

For peer review only

For peer review only - http://bmjopen.bmj.com/site/about/guidelines.xhtml

Trends showed an increase in proportions of patients admitted with stroke compared to other conditions over the 3 years from 9.4% in 2017 to 11.9% in 2018 and 14.5% in 2019 **(Figure 1).**

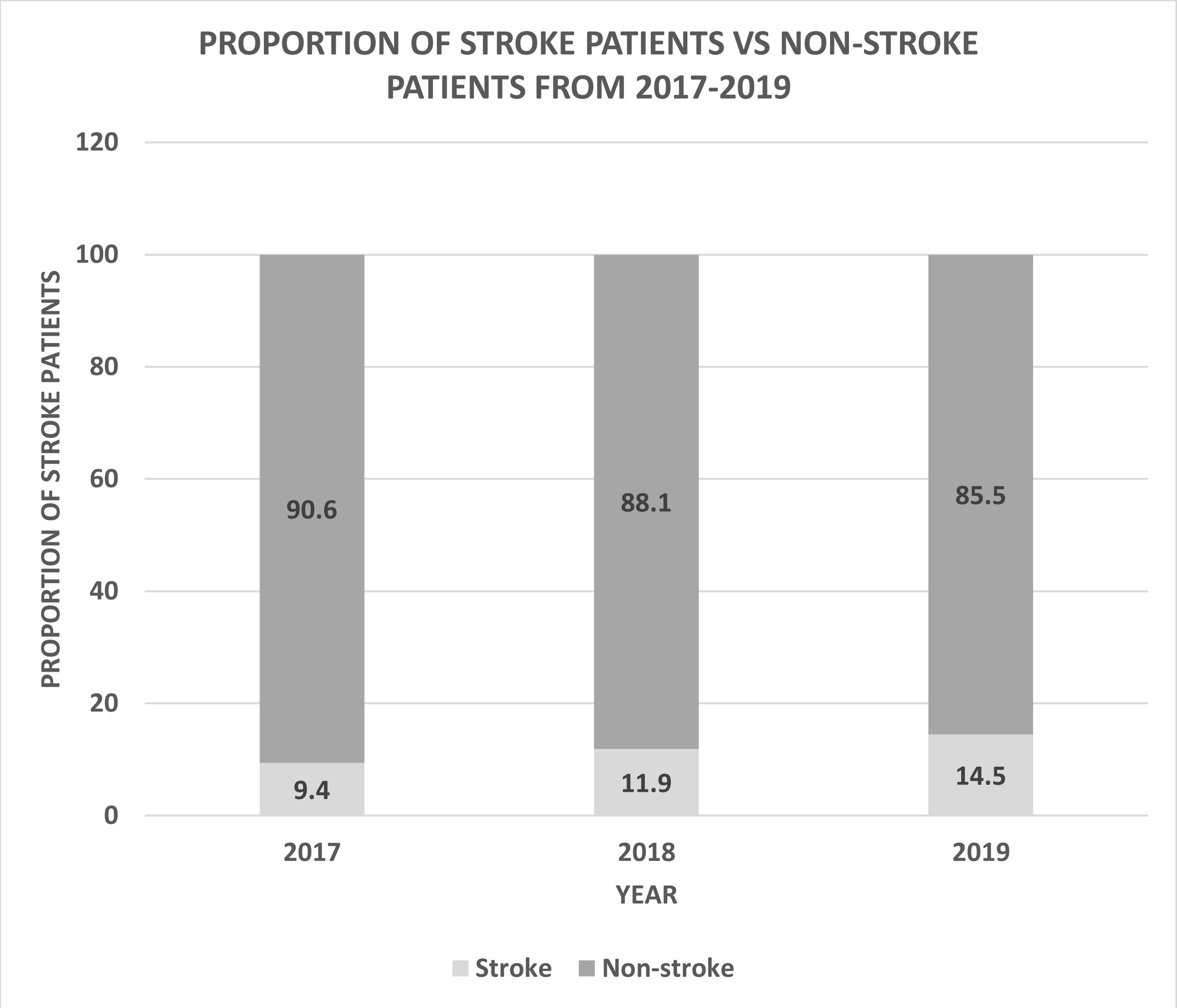

Figure 1: Proportion of patients admited with stroke compared to admission of non-stroke patients

While the proportion of deaths of non-stroke patients decreases from 35.2%, 34.6% to 30.2% as from 2017, 2018 and 2019 respectively**,** the proportion of stroke deaths increased, with 11.9%, 16.7% and 19.8% for 2017, 2018 and 2019, respectively (**Figure 2).**

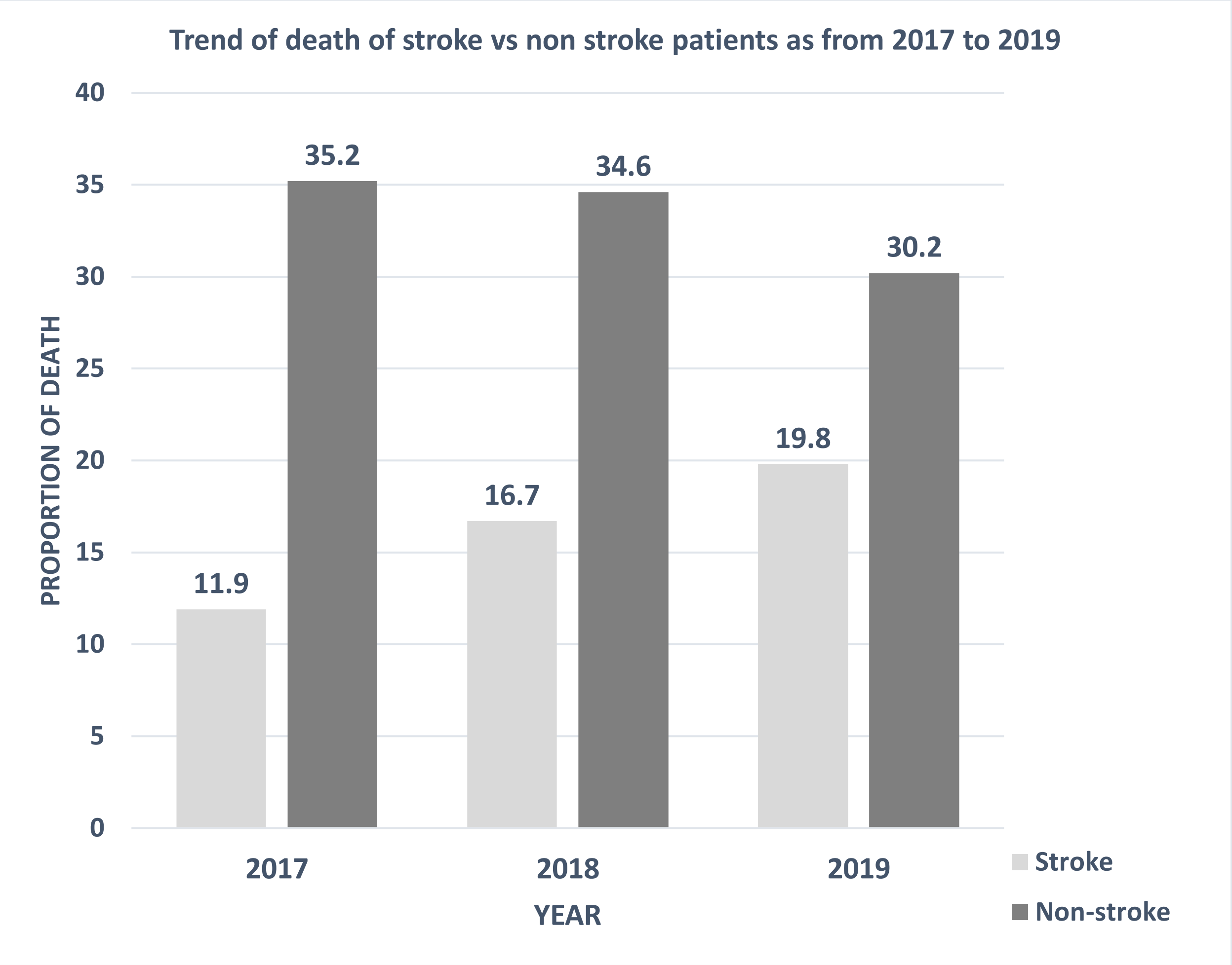

Figure 2: Proportions of deaths for stroke vs non-stroke patients for 2017-2019

The number of stroke patients increased over the three years consecutively from 222, 292 to 458, revealing that between 2018 and 2019 number of stroke patients almost doubled **(Supplementary Figure 1).**

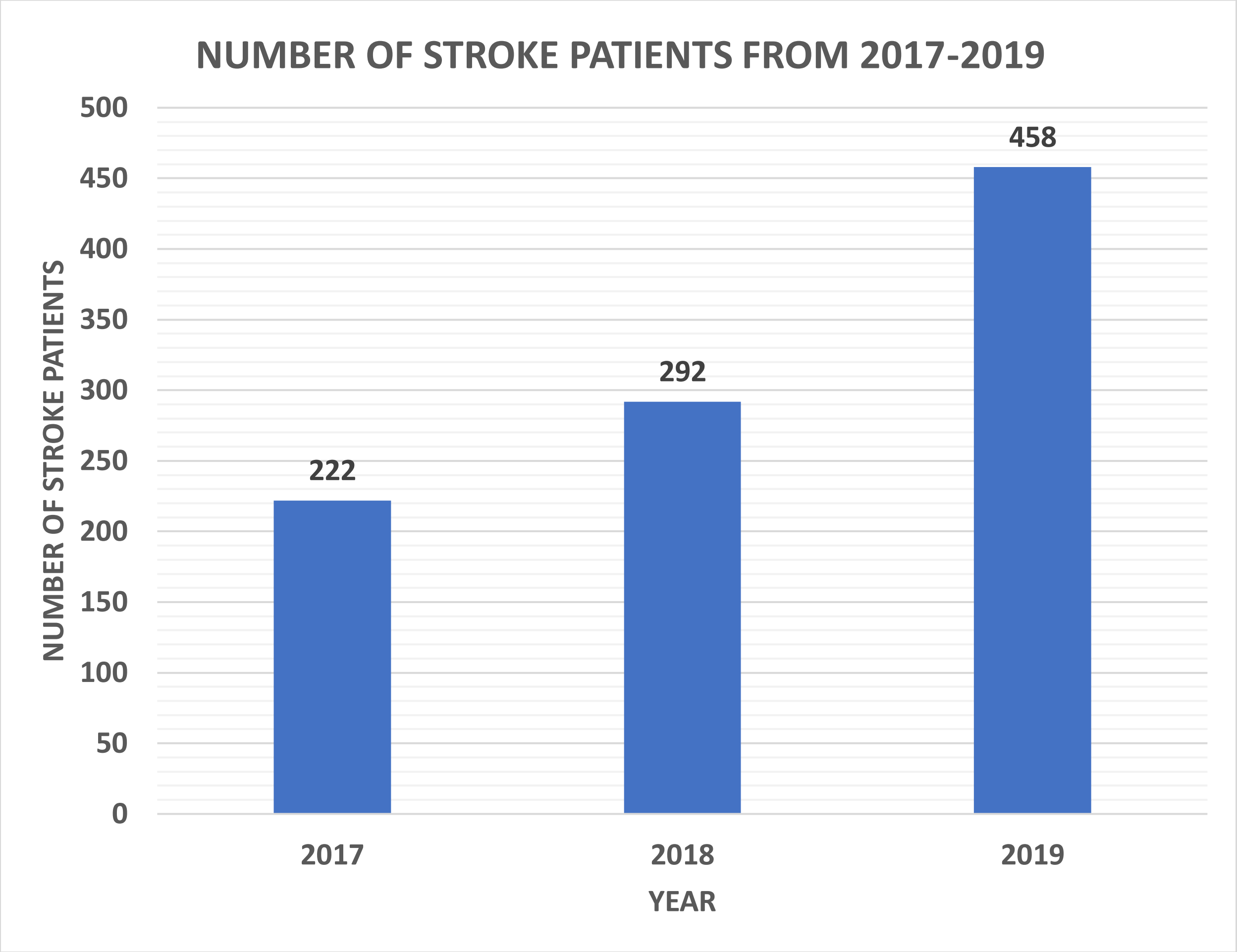

Supplementary Figure 1: Number of stroke admissions for 2017 to 2019

From 2017 to 2019 the number of deaths for non-stroke patients decreases from 506, 498 to 435 respectively. However, for the same three years the number of deaths for stroke patients increases from 60, 83 to 86 consecutively **(Supplementary Figure 2).**

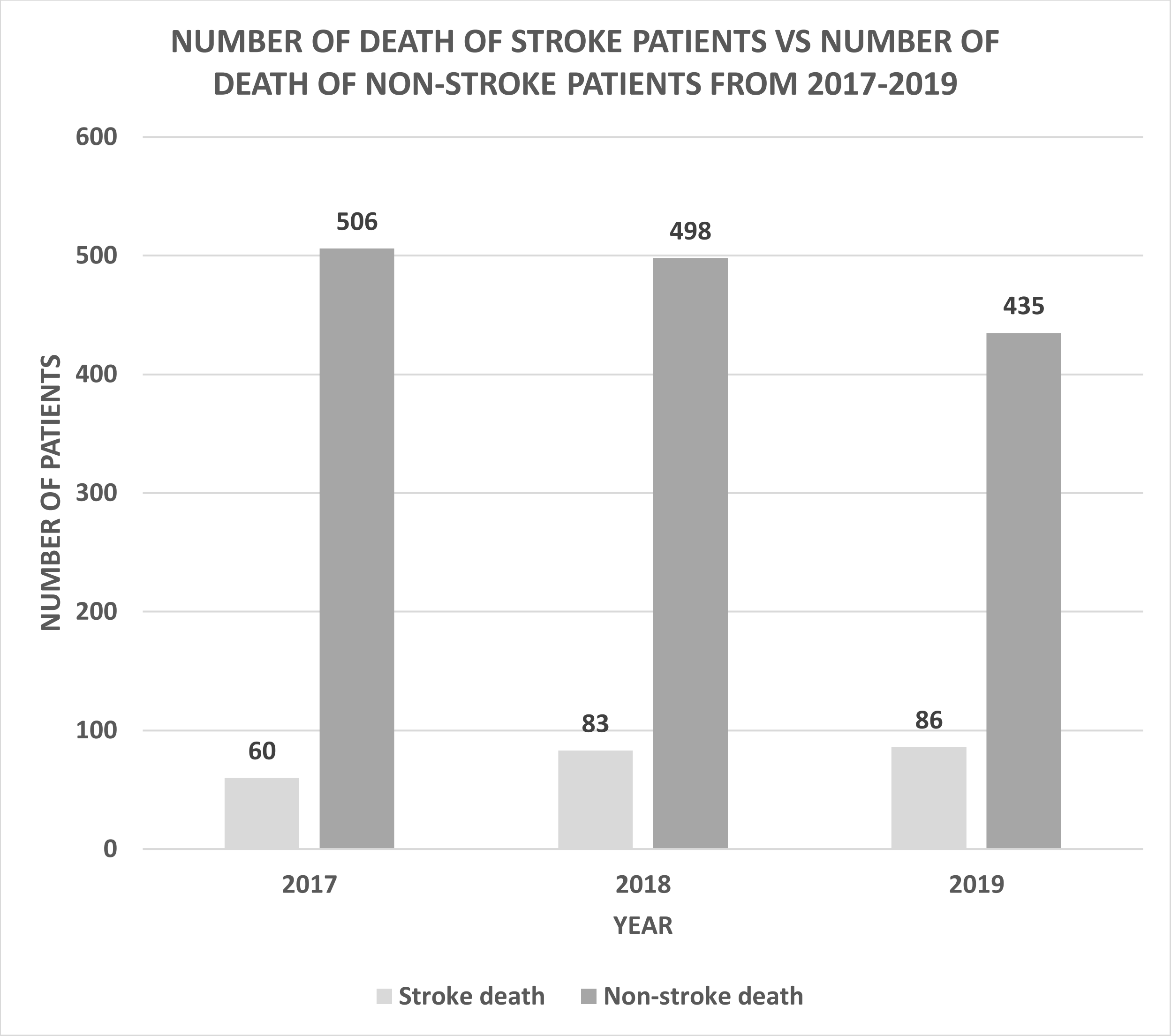

Supplementary Figure 2: Number of deaths for stroke patients Vs non-stroke patients for 2017-2019

Among all stroke patients identified, 229 (23.6%) died, while 458 (47.1%) of stroke patients stayed in hospital for more than thirty days **(Supplementary Table 1).**

Supplementary Table 1. Outcomes among stroke patients hospitalized at KCMC from 2017-2019.

Variables	Stroke		
	**Yes n (%) n=972**	**No n (%) n=7004**	***p*-value**
**Duration of Hospital stay (days)**			
0-7	202 (20.8)	1483 (21.2)	0.609
8-14	68 (7.0)	503 (7.2)	
15-21	110 (11.3)	872 (12.5)	
22-29	132 (13.6)	1009 (14.4)	
>30	458 (47.1)	3117 (44.5)	
Missing	2 (0.2)	20 (0.3)	
**Discharge status**			
Dead	229 (23.6)	1210 (17.3)	<0.001
Alive	742 (76.3)	5788 (82.6)	
Missing	1 (0.1)	6 (0.1)	

For peer review only

STROBE Statement—Checklist of items that should be included in reports of ***cross-sectional studies***

	Item No	Recommendation	Page number
**Title and abstract**	1	(*a*) Indicate the study's design with a commonly used term in the title or the abstract	2
		(*b*) Provide in the abstract an informative and balanced summary of what was done and what was found	2-3
**Introduction**			
Background/rationale	2	Explain the scientific background and rationale for the investigation being reported	4-5
Objectives	3	State specific objectives, including any prespecified hypotheses	6
**Methods**			
Study design	4	Present key elements of study design early in the paper	7
Setting	5	Describe the setting, locations, and relevant dates, including periods of recruitment, exposure, follow-up, and data collection	7-8
Participants	6	(*a*) Give the eligibility criteria, and the sources and methods of selection of participants	8
Variables	7	Clearly define all outcomes, exposures, predictors, potential confounders, and effect modifiers. Give diagnostic criteria, if applicable	8
Data sources/ measurement	8*	For each variable of interest, give sources of data and details of methods of assessment (measurement). Describe comparability of assessment methods if there is more than one group	8
Bias	9	Describe any efforts to address potential sources of bias	8
Study size	10	Explain how the study size was arrived at	n/a
Quantitative variables	11	Explain how quantitative variables were handled in the analyses. If applicable, describe which groupings were chosen and why	8
Statistical methods	12	(*a*) Describe all statistical methods, including those used to control for confounding	8
		(*b*) Describe any methods used to examine subgroups and interactions	n/a
		(*c*) Explain how missing data were addressed	n/a
		(*d*) If applicable, describe analytical methods taking account of sampling strategy	n/a
		(*e*) Describe any sensitivity analyses	n/a
**Results**			
Participants	13*	(a) Report numbers of individuals at each stage of study—eg numbers potentially eligible, examined for eligibility, confirmed eligible, included in the study, completing follow-up, and analysed	n/a
		(b) Give reasons for non-participation at each stage	n/a
		(c) Consider use of a flow diagram	n/a
Descriptive data	14*	(a) Give characteristics of study participants (eg demographic, clinical, social) and information on exposures and potential confounders	10-15
		(b) Indicate number of participants with missing data for each variable of interest	n/a
Outcome data	15*	Report numbers of outcome events or summary measures	n/a
Main results	16	(*a*) Give unadjusted estimates and, if applicable, confounder-adjusted estimates and their precision (eg, 95% confidence interval). Make clear which confounders were adjusted for and why they were included	10-15

For peer review only

		(*b*) Report category boundaries when continuous variables were categorized	n/a
		(*c*) If relevant, consider translating estimates of relative risk into absolute risk for a meaningful time period	n/a
Other analyses	17	Report other analyses done—eg analyses of subgroups and interactions, and sensitivity analyses	n/a
**Discussion**			
Key results	18	Summarise key results with reference to study objectives	
Limitations	19	Discuss limitations of the study, taking into account sources of potential bias or imprecision. Discuss both direction and magnitude of any potential bias	16
Interpretation	20	Give a cautious overall interpretation of results considering objectives, limitations, multiplicity of analyses, results from similar studies, and other relevant evidence	10-16
Generalisability	21	Discuss the generalisability (external validity) of the study results	16
**Other information**			
Funding	22	Give the source of funding and the role of the funders for the present study and, if applicable, for the original study on which the present article is based	17

*Give information separately for exposed and unexposed groups.

Note: An Explanation and Elaboration article discusses each checklist item and gives methodological background and published examples of transparent reporting. The STROBE checklist is best used in conjunction with this article (freely available on the Web sites of PLoS Medicine at http://www.plosmedicine.org/, Annals of Internal Medicine at http://www.annals.org/, and Epidemiology at http://www.epidem.com/). Information on the STROBE Initiative is available at www.strobe-statement.org.

For peer review only
